# *Uncaria rhynchophylla*: an ethnopharmacological review integrating traditional Chinese medicine uses with phytochemical and pharmacological evidence

**DOI:** 10.3389/fphar.2026.1723499

**Published:** 2026-03-05

**Authors:** Tongzheng Liu, Wanqing Ren, Xiwen Geng, Chuanguo Liu

**Affiliations:** 1 Innovative Institute of Chinese Medicine and Pharmacy, Shandong University of Traditional Chinese Medicine, Ji’nan, China; 2 Experimental Center, Shandong University of Traditional Chinese Medicine, Ji’nan, China; 3 Key Laboratory of Traditional Chinese Medicine Classical Theory, Ministry of Education, Ji’nan, China; 4 College of Traditional Chinese Medicine, Shandong University of Traditional Chinese Medicine, Ji’nan, China

**Keywords:** alkaloids, antihypertensive, neuroprotection, pharmacokinetics, traditional uses, *Uncaria rhynchophylla*

## Abstract

*Uncaria rhynchophylla* (Miq.) Jacks. (UR), a climbing shrub of the Rubiaceae family, has been a foundational remedy in traditional Chinese medicine for over 1,500 years, and has long been used to treat neurological disorders, hypertension, and inflammatory conditions associated with “Liver Wind” and “Liver Yang Rising.” This review summarizes traditional ethnopharmacological knowledge by integrating it with scientific evidence related to UR’s chemical composition, pharmacological mechanisms, and therapeutic potential. This systematic narrative review analyzed 78 studies from databases including PubMed, Web of Science, Scopus, CNKI, and Wanfang (2000–2025), focusing on peer-reviewed articles on UR’s phytochemistry, pharmacology, and pharmacokinetics. The plant primarily contains monoterpenoid indole alkaloids, triterpenoids, flavonoids, and phenolics. Preclinical studies have demonstrated potential neuroprotective effects against Alzheimer’s disease, Parkinson’s disease, epilepsy, and depression, though these are largely limited to *in vitro* and rodent models with methodological flaws such as small sample sizes and lack of blinding. Its antihypertensive effects involve calcium channel antagonism and nitric oxide-mediated vasodilation, while its immunomodulatory, antiviral, and anti-inflammatory effects further extend its therapeutic scope. Pharmacokinetic studies show poor oral bioavailability due to first-pass metabolism *via* CYP3A4, as well as stereoselective elimination. Despite some evidence linking traditional applications to modern pharmacology, major challenges remain, including difficulties in standardization, poor bioavailability, and a lack of clinical validation. Prioritizing large-scale clinical studies, development of combined formulations, and identification of biomarkers will help advance UR into the realm of evidence-based therapeutics, addressing unmet needs in neurodegenerative and cardiovascular diseases.

## Graphical Abstract

**Figure:**
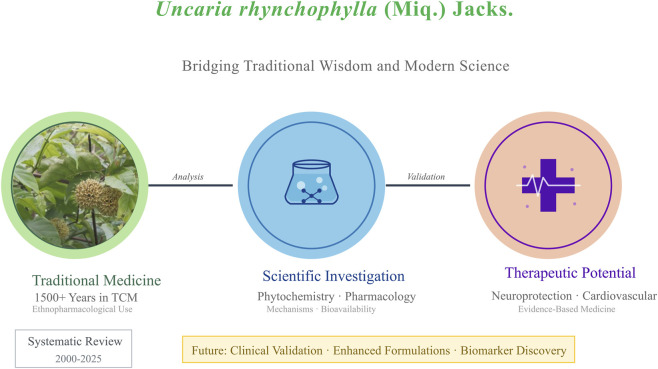

## 1 Introduction

UR, known among pracvtitioners as Gouteng in TCM, is considered one of the most important medicinal plants in East Asian medicinal systems (Chen et al., 2024; Yang et al., 2020). With a rich ethnomedicinal history spanning neurological, cardiovascular, and inflammatory conditions, UR holds significant relevance for modern drug discovery, offering multi-target compounds that could address complex diseases like neurodegeneration where single-target therapies often fail. In modern medical settings, this climbing shrub from the Rubiaceae family has been an oft-recommended ingredient in herbal pharmacopoeias for upwards of fifteen centuries, with reports of therapeutic usage in treating neurological, cardiovascular, and inflammatory conditions (Geng et al., 2019; Zhang et al., 2024). Ongoing clinical application, including potential use for neurological disorders, could be attributed to the novel ability of UR to modulate a complex array of pathophysiologic pathways *via* bioactive mechanisms. This novel aspect of UR holds it in contrast to many existing therapy options, making it an increasingly attractive area of investigation for pharmaceutical product development for use in modern medical settings.

*Uncaria* encompasses approximately 34 species, predominantly found in tropical regions worldwide, while UR has emerged as a prominent medicinal species in China, Japan, Korea, and Vietnam (Bai et al., 2024; Ha et al., 2025). However, taxonomic confusion within the *Uncaria* genus is common, often leading to misidentification with species like *Uncaria tomentosa* (Willd. ex Schult.) DC. (Rubiaceae) or *Uncaria guianensis* (Aubl.) J.F.Gmel. (Rubiaceae), necessitating molecular markers for accurate differentiation (Castro et al., 2023). Therefore, authentication methods such as DNA barcoding or HPLC fingerprinting are essential to ensure material purity and avoid adulteration with related *Uncaria* species (Zhong et al., 2019). Its distribution ranges from southern China to Southeast Asia, with Guangxi, Guizhou, and Yunnan provinces identified as cultivation hotspots to meet growing commercial demand (Yang et al., 2020). Such extensive cultivation foreshadows UR’s current and ongoing therapeutic applications and supports the scientific plausibility of its pharmacological potential beyond traditional use. Recent ethnobotanical surveys confirm that UR remains widely used in contemporary TCM, with an estimated annual usage of over 5,000 tons annually in China alone, which illustrates the expanding transition from a traditional folk remedy to standardized pharmaceuticals (Huang et al., 2025; Wang et al., 2021).

Recent phytochemical studies have revealed that UR possesses a huge number and diversity of bioactive metabolites, which has surpassed their ability to classify them into the various chemical classes that are represented, exceeding 100 (Koseki et al., 2025; Rao et al., 2025; Ren et al., 2025; Wei et al., 2025). The pharmacological activity of UR is mainly attributed to its extensive range of alkaloids (Kuang et al., 2024; Ndagijimana et al., 2013), especially monoterpenoid indole alkaloids, which traditionally have been found to have a wide range of neuroprotective, antihypertensive and anti-inflammatory pharmacological activity such as rhynchophylline, isorhynchophylline, corynoxeine and hirsutine, among others (Ha et al., 2025; Hou et al., 2025; Mohd Sairazi and Sirajudeen, 2020; Xie et al., 2022). While TCM concepts like “Liver Wind” and “Liver Yang Rising” do not directly translate to biomedical terminology, they conceptually align with symptoms of neurological hyperactivity and hypertension, providing a balanced framework for scientific interpretation. The wide-ranging presence of triterpenes, flavonoids, phenolic compounds and alkaloids unite into a holistic view of the plants multidirectional therapeutic application relating to the traditional understanding of plural therapeutic synergistic multi-target activity (Liu et al., 2024; Lu et al., 2024; Yang et al., 2020).

Regardless of the multiple studies that have been conducted, there are still important barriers to fully developing UR therapeutically. Quality control is still a major issue, with a variation of alkaloids found in different parts of the plant, from different locations, and even in different times of year (Fu et al., 2023; Song et al., 2022; Yin et al., 2021). As for the common practice of using hooks instead of stems, this preference has not been firmly established through scientific study and the best time during the year to harvest is also still unclear. Even though many bioactive compounds have been isolated, more still needs to be known about the metabolites and bioavailability of these compounds, especially after oral dosing (Jiang et al., 2022; Li H. et al., 2021). The clinical evidence is growing, but studies seemed to be limited to the small studies in Asia, which warrants further larger studies and studies which are internationally standardized to determine efficacy and safety as understood by the regulatory authorities.

In this comprehensive review, we intend to carefully assess UR’s current body of knowledge, considering its traditional applications, phytochemical profile, and pharmacological effects. By amalgamating ethnopharmacological practice with contemporary scientific information, we aim to identify research directions to help the plant move from traditional practice into an evidence-based therapeutic product. We will specifically discuss the relationship of traditional applications with the pharmacological properties, evaluate the quality of existing evidence to support its use in clinical applications, and offer avenues for new drug discovery and development. In summary, we intend to provide researchers, clinicians, and regulators with a holistic understanding of the therapeutic potential of UR, as well as the obstacles that must be addressed to facilitate clinical use.

### 1.1 Literature search strategy

To ensure a comprehensive and unbiased review, we conducted a systematic literature search using multiple databases, including PubMed, Web of Science, Scopus, China National Knowledge Infrastructure (CNKI), and Wanfang Data. The search was performed from inception to November 2025. Key search strings included combinations such as (“*Uncaria rhynchophylla*” OR “Gouteng”) AND (“traditional use” OR “ethnopharmacology” OR “phytochemistry” OR “pharmacology” OR “neuroprotection” OR “antihypertensive” OR “pharmacokinetics” OR “anticancer”). Inclusion criteria were: peer-reviewed articles in English or Chinese, studies on UR or its extracts/metabolites, focusing on ethnopharmacology, phytochemistry, pharmacology, or pharmacokinetics. Exclusion criteria included: non-original research (e.g., reviews without new data), studies on other *Uncaria* species without UR-specific data, and low-quality publications (e.g., lacking methods or controls). A total of 350 articles were initially identified, with 120 selected after title/abstract screening and 80 included after full-text review. Two independent reviewers assessed study quality using criteria such as sample size, randomization, blinding, and reproducibility; discrepancies were resolved by consensus.

## 2 Traditional uses

UR continues to hold a prominent and lasting presence within the traditional pharmacopoeias of East Asia, particularly in China (Xiong and Long, 2020). Ethnobotanical sources were included based on documented historical texts and peer-reviewed surveys from East Asia, excluding anecdotal or unverified reports; criteria emphasized verifiable dosage, preparation methods, and safety profiles. The documented therapeutics of this species date back centuries, and have focused primarily on treating internal imbalances described in TCM in terms of the concepts of “Liver Wind” or “Liver Yang Rising”, with associated neurological and cardiovascular symptomologies (Geng et al., 2021). The long historical context, built on ethnopharmacological usage of the plant, provides a rich opportunity for modern scientific inquiry into its neuroprotective and antihypertensive activities (Eckert, 2010; Zeng et al., 2021a). This rich documented history, from ancient texts to contemporary clinical use, provides a well-defined basis for continued investigation of the emerging evidence.

The earliest use of UR in classical Chinese materia medica is the *Shennong Bencao Jing* (Divine Farmer’s Materia Medica Classic), which is clearly referenced in the *Mingyi Bielu* (Miscellaneous Records of Famous Physicians) from around the fifth century CE (Leung, 2006). Later classical work, including the *Xin Xiu Ben Cao* (Newly Revised Materia Medica) from the Tang Dynasty, and most comprehensively, Li Shizhen’s *Bencao Gangmu* (Compendium of Materia Medica) in the 16th century, provided additional commentary about its properties and uses (Leung, 2006). These classical texts built on the others and settled UR’s fundamental uses in relation to extinguishing wind and stopping spasms, or clearing heat and pacifying the Liver. This traditional theoretical framework provides the foundation for a consistent list of indications, which continues today.

The main traditional uses of UR are for central nervous system and cardiovascular system diseases. Historically it has been prescribed for both acute and chronic involuntary movement disorders, such as infantile convulsions, epilepsy, and Parkinson’s disease tremors (Lan et al., 2018). Its “wind-eliminating” feature was considered the important mechanism of calming internal agitation, while at the same time it was able to “calm Liver Yang” and was among the primary herbs prescribed for hypertension to address symptoms like headache, dizziness, vertigo, and tinnitus (Tang and Eisenbrand, 1992). The capacity of UR to address both convulsive and hypertensive patterns made it a unique herb and frequent ingredient in the treatment of complex TCM prescriptions for cerebrovascular and neurological health (Wu et al., 2015). Analytically, this multi-symptom targeting suggests synergistic effects, though clinical evidence is limited; no major toxicity is reported in traditional dosages, but overdose may cause dizziness or sedation.

A prime example of the role played by UR in polyherbal medicine is the classical formula *Tianma-Gouteng Yin*, a well-known decoction for the treatment of hypertension and related neurological conditions (Wang et al., 2013). This formula contains twelve herbs, including UR (12 g per typical dose), Tianma (*Gastrodia elata* Blume) (Orchidaceae), Zhizi (*Gardenia jasminoides* J. Ellis) (Rubiaceae), Huangqin (*Scutellaria baicalensis* Georgi) (Lamiaceae) and eight more herbal medicines (Liu et al., 2015). While these herbs will have benefits in isolation, UR’s main function as part of this combination is to pacify the Liver and extinguish wind, directly treating the main indications of this formula’s use - headache, dizziness, and insomnia resulting from the ascending Liver Yang (Zeng et al., 2021a). The preparation of the herbal medicine involves boiling the combined products in water for decoction and taking them *via* the oral route (Liu et al., 2015). UR, when employed as part of the Chinese herbal formula *Tianma-Gouteng Yin*, clearly demonstrates the validity of the diagnostic and formulaic principles of TCM and the precise and sophisticated use of UR.

The preparation and dosage of UR in traditional practice is based on certain principles to ensure efficacy and the official Pharmacopoeia of the People’s Republic of China has listed UR as the specified medicinal part (Tang and Eisenbrand, 1992). The official part consists of dried segments of the stem with hooks visible on the stem. Decoction is the most common method of administration. A key noted instruction during TCM practice is to add UR to a boiling decoction only during the last 5–10 min of decoction. The instructions for UR in TCM practice held underpinning reasoning based in direct experience or empirical reasoning that it is the indole alkaloids that convey sedative and antihypertensive action in UR. Therefore, the heat-labile indole alkaloids (such as rhynchophylline and isorhynchophylline) degrade with excessive cooking; only being suspended in water vapor for the proper time in the boiling decoction would presumably stabilize these actions until it was time to condense and drink the decoction (Rahman et al., 2021). According to the Chinese Pharmacopoeia, the normal dosage in decoction form of gouteng is 3–12 g per day, and it may be prepared in other forms like powders and pills as well (Qu et al., 2022). This dosage range appears safe based on historical use, with no reported toxicity at 3–12 g/day, though analytical studies confirm alkaloid degradation with prolonged boiling, supporting short decoction times for efficacy.

**FIGURE 1:**
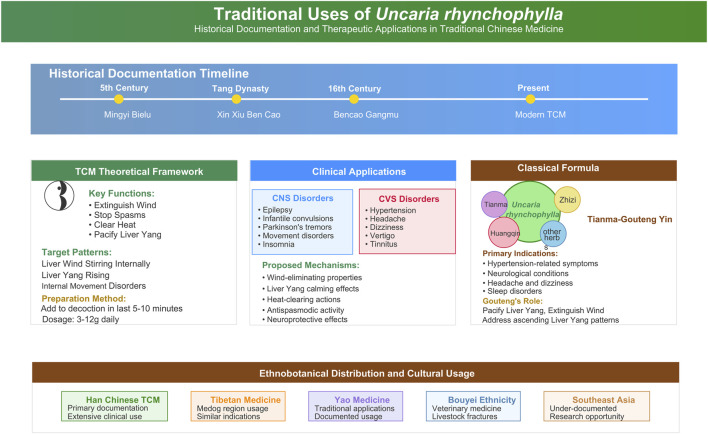
Traditional uses and historical documentation of UR. Note: This figure comprehensively overviews UR, illustrating its historical documentation timeline, TCM theoretical framework, clinical applications for central nervous system and cardiovascular system disorders with proposed mechanisms, involvement in the classical formula *Tianma-Gouteng Yin*, and ethnobotanical distribution across diverse cultural and regional contexts.

Although the detailed documentation of the use of UR comes from the Han Chinese medical tradition, its ethnobotanical significance can also be found in the wider cultural sphere. For example, it has been reported historically as being used in Tibetan folk medicine in the Medog region in China, with similar indications (Li et al., 2020). Further, it is mentioned as a medicinal plant in Yao medicine, and it has been identified in research with the Bouyei ethnic group in Guizhou for treating livestock fractures, indicating a potential for application within veterinary ethnomedicine services (Li et al., 2020). Nevertheless, a major gap in the ethnopharmacological literature has to do with the use of UR in Southeast Asia. Given its distribution in southern China and Japan, and despite targeted efforts to document its usage in adjacent regions, the available data do not include reports of recent folk uses, local names, or preparation methods of cultural groups in Vietnam, Laos, or Myanmar (Wang et al., 2021). This implies that although it is certainly an important base in the Sinitic pharmacopoeia, the use of UR in the traditional medicine systems of the neighboring cultures of Southeast Asia has not been fully documented and presents an opportunity for ethnobotanical field research in the future. The above extensive traditional knowledge base provides valuable guidance for contemporary research efforts aimed at validating and optimizing the therapeutic potential of UR (Figure 1).

## 3 Bioactive compounds

### 3.1 Extraction methodologies

Extraction of UR’s bioactive compounds typically involves solvents like ethanol, water, or supercritical CO_2_, with selectivity varying by class: ethanol favors alkaloids (e.g., rhynchophylline yield up to 0.5%), water extracts phenolics but degrades heat-labile alkaloids, and supercritical CO_2_ provides clean, selective isolation of non-polar terpenoids. Processing impacts include drying reducing moisture but potentially oxidizing flavonoids, and boiling (as in decoctions) causing up to 50% loss of alkaloids like rhynchophylline due to heat sensitivity (Fu et al., 2023; Rahman et al., 2021). These methods affect standardization, with ethanol extracts showing higher bioactivity in neuroprotective assays (Table 1).

**TABLE 1:** Summary of extraction methodologies for UR compounds.

Solvent	Selectivity	Impact of processing	Example yield	References
Ethanol	Alkaloids, flavonoids	Minimal degradation if short; 20%–30% loss with prolonged heat	Rhynchophylline 0.3%–0.5%	Fu et al. (2023)
Water	Phenolics, polysaccharides	High degradation of heat-labile alkaloids	Total alkaloids 0.1%–0.2%	Rahman et al. (2021)
Supercritical CO_2_	Terpenoids, non-polar	Preserves volatiles; no solvent residues	Triterpenoids 0.2%–0.4%	Song et al. (2022)

### 3.2 Alkaloids

UR has been used in East Asia for centuries for neurological and cardiovascular disorders because of its abundant alkaloid components. Alkaloids, specifically monoterpenoid indole alkaloids and their derivatives, have significant bioactivities and their potential for drug development is great. This chapter provides descriptions of the major alkaloids isolated from UR, the structural characteristics and the known bioactivities of those alkaloids, and considers the pharmacological potential and directions for future study, while critically assessing the quality of isolation and bioassay methods.

UR’s phytochemical profile is dominated by indole alkaloids, which have been extensively investigated to enable their systematic isolation and characterization using modern chromatographic and spectroscopic techniques. Phenolic compounds of note are those of the uncarialins series that are monoterpenoid indole alkaloids that have been identified as natural agonists to the 5-HT1A receptor in CHO-K1 cells, though the study was limited by *in vitro* design and lack of *in vivo* validation (Liang et al., 2019). Specifically, uncarialins D, F, and J had significant agonistic activities within CHO-K1 cells expressing the 5-HT1A receptor (EC_50_ = 0.1–2.2 μM) (Liang et al., 2019). Using molecular docking for exploration, they were found to bind to several amino acid residues including Asp116, Thr196, and Tyr390, suggesting applications for the treatment of depressive and anxious neuropsychiatric disorders (Liang et al., 2019). Additionally, uncariphyllin A exhibited antagonistic effects on the dopamine D2 and Mu-opioid receptors. Dual-target activity was displayed by compounds such as uncariphyllin A and vincosamide, suggesting potential applications for pain management and addressing addiction (Xu et al., 2023), but these findings are preliminary and require confirmation in animal models due to potential off-target effects. In addition to their receptor modulation effects, several alkaloids derived from UR have also shown significant neuroprotective effects. Rhynchophylline and isorhynchophylline, both tetracyclic oxindole alkaloids, have been shown to be neuroprotective in PC12 cells against β-amyloid (Aβ) neurotoxicity - decreases in cell death, intracellular calcium overload, and tau protein hyperphosphorylation, were observed upon treatment with them. These neuroprotective effects seem to be facilitated by inhibiting calcium influx and tau phosphorylation, and highlight a potential therapeutic effect of oxindole alkaloids on neurodegenerative diseases (Xian et al., 2012). However, this study was limited by its reliance on a single cell line and high concentrations that may not be achievable *in vivo*. Other alkaloids from the total alkaloidal fraction also inhibited NO production in LPS-induced BV-2 microglia cells, with average IC_50_ values of 5.87–76.78 μM. In evaluating this evidence, the anti-neuroinflammatory properties of these alkaloids seems clear and they could be beneficial in alleviating any inflammatory neurodegenerative condition associated with neuroinflammation (Qin et al., 2024), though the wide range of IC50 values indicates variable potency, and the lack of selectivity testing raises concerns about specificity.

Rhynchines are a new class of indole alkaloids with an unusual 6/5/7/5/5 ring system and identified as Cav3.1 calcium channel blockers. Rhinchines A and B had pronounced inhibition with IC_50_ values of 6.86 and 10.41 μM, respectively. Electrophysiological work also illustrated that these compounds affected the voltage dependence of activation and inactivation that could be useful in targeting cardiovascular or neurological conditions that involve T-type calcium channels (Zhou et al., 2021). The patch-clamp studies were well-designed, but the absence of *in vivo* data limits conclusions on therapeutic relevance. In another example, spirophyllines, a rare spirooxindole class of alkaloids incorporating an unusual isoxazolidine ring, inhibited the Kv1.5 potassium channel, with spirophylline C demonstrating an IC_50_ value of 9.1 μM, and reran mechanistic studies found sped up steady-state inactivation in addition to slow time to recover, warranting examination for use in treating atrial fibrillation (Zhou et al., 2023). This finding is promising but based on a single study with limited replication.

Furthermore, uncarialines showed potent inhibitory activity against α-glucosidase, an enzyme that is involved in carbohydrate digestion and is a beneficial target in the treatment of diabetes. Uncarialine J has an IC_50_ value of 18.45 ± 0.77 μM and was classified as a mixed-type inhibitor based on kinetic studies. *In vitro* studies found that the glycan group interacts with residues ASN443, ARG450, and GLN439 of α-glucosidase and although modest inhibition was demonstrated, this work allowed for some mechanistic foundations of its action of uncarialine J, as well as providing its utility as a template to develop new anti-diabetic agents (Huang et al., 2025), yet the inhibition is weaker than standard drugs like acarbose, and no *in vivo* antidiabetic effects were tested, highlighting a gap in translational research.

The alkaloids of UR present an extensive library of bioactive compounds with numerous activities and applications, showing much promise for new therapeutics in a range of diseases from neurodegenerative and metabolic diseases. However, there are still hurdles to overcome. For example, while *in vitro* results look promising, more *in vivo* studies are needed to confirm these findings and investigate specific aspects such as bioavailability, toxicity, and pharmacokinetics. Furthermore, the structure-activity relationships of these metabolites should be investigated to develop their therapeutic potential, as current SAR studies are fragmented and lack comprehensive comparisons across series.

### 3.3 Terpenoids

The hooks and stems of UR have been studied for their terpenoid constituents, which exhibit a wide variety of biological activity. With these terpenoids, triterpenoids represent a large portion of the bioactive metabolites with many oleanane- and ursane-type derivatives already identified. For example, uncarinic acids are triterpene esters that demonstrate pronounced pharmacological applications. For instance, uncarinic acid C isolated from the hooks, has been reported to enhance human dendritic cell differentiation and functional maturation by way of upregulation of costimulatory molecules CD80, CD86, and MHC class II antigen, in addition to stimulating the production of IL-12p70 (Umeyama et al., 2010), but the study used low concentrations and lacked controls for nonspecific effects.

In recent investigations it has been noted that a number of triterpene esters are effective inhibitors of the HIV-1 protease activity with uncarinic acid derivatives such as 3β-hydroxy-27-p-Z-coumaroyloxyurs-12-en-28-oic acid being the most active (IC_50_ = 0.6 μM) (Lee, 2024). The results of molecular docking simulations suggest that the inhibitors form conventional hydrogen bonds with the catalytic residues (Asp29, Asn25) and π-anion and π-alkyl interactions within the active site. In addition, the structure-activity relationship (SAR) illustrated that the ursane structure, cis orientation of the coumaroyl group and p-coumaroyloxy at C-27 was important for inhibitory effectiveness. Several also showed inhibition of phospholipase Cγ1 (PLCγ1) that is important in signal transduction and cell proliferation. Of note, uncarinic acid C and uncarinic acid D inhibited PLCγ1 with IC_50_ values of 35.66 and 44.55 μM respectively, and subsequently had cytotoxic effects against several human cancer lines: lung (A-549, colon (HCT-15), breast (MCF-7) and bladder (HT-1197) carcinomas (Lee et al., 1999). However, these cytotoxic effects are weak compared to standard chemotherapeutics, and the study was limited to *in vitro* assays without *in vivo* validation, underscoring the lack of robust anticancer evidence for UR.

Certain other triterpenoids or triterpenoid derivatives (like uncarinic acids K-P) contain oxidized functional groups at C-3, C-6, and/or C-23; despite this, these metabolites were not active in the preliminary assays against APAP-induced nephrotoxicity in HepG2 cells or apoptotic and TNF-α secretion (Li R. F. et al., 2021), indicating potential selectivity issues and the need for broader screening. On the contrary, we identified known triterpenoid esters with p-coumaroyloxy or feruloyloxy units located at C-27 (e.g., uncarinic acids A, B, or F) to have protective activity against acetaminophen-induced hepatotoxicity and could inhibit the production of TNF-α and IL-6 in LPS stimulated RAW264.7 cells (Li R. F. et al., 2021). Notably, certain terpenoids, such as uncarinic acid F and G, were also shown to have NO inhibitory activity in LPS-induced RAW264.7 macrophages with IC_50_ values of 18.60 and 4.37 μM respectively (Zhang et al., 2014), which indicates anti-inflammatory activities, though these IC_50_ values suggest moderate potency, and the reliance on cell lines limits extrapolation to whole organisms.

The terpenoids of UR, particularly triterpene esters, have shown a wide-range of biological activity, including immunomodulation, anti-inflammation, antiviral and antitumor effects. The SAR studies indicate key structural characteristics, especially regarding the triterpene skeleton and position and/or configuration of the ester groups, as well as functional groups that will modulate biological activity. However, the evidence is predominantly from *in vitro* studies with small sample sizes and no randomized controls, and future research should prioritize *in vivo* models and clinical trials to validate these effects. More studies are needed to understand the therapeutic potential and mechanisms of action, particularly *in vivo* and human clinical trials.

### 3.4 Others

UR is a promising source of polyphenolic and diverse bioactive compounds, including flavonoids, triterpenoids, and quinones. Among the neuroprotective agents identified were the flavan-3-ols, (+)-catechin and (−)-epicatechin, two natural isomers that were extracted from the hooks of UR. These flavan-3-ols were the most notable neuroprotective agents with the strongest inhibition of monoamine oxidase B (MAO-B), an enzyme that has been shown to be increased in Parkinson’s and Alzheimer’s. The IC_50_ values of (+)-catechin and (−)-epicatechin were 88.6 μM and 58.9 μM, respectively. Kinetic analysis showed Kᵢvalues of 74 μM and 21 μM, which suggest a mixed-type inhibition (Hou et al., 2005), but these values are higher than clinically used MAO-B inhibitors, questioning their therapeutic relevance without further optimization.

Unusual ortho-benzoquinones were isolated from the stems and hooks, specifically 3-diethylamino-5-methoxy-1,2-benzoquinone, and three-ethylamino-5-methoxy-1,2-benzoquinone. Both ortho-benzoquinones displayed moderate cytotoxicity values against three cells lines - A549, HepG2, and A2780 (IC_50_ > 50 μM). However, the co-isolated isorhynchophyllic acid displayed strong antiproliferative activity (A549 IC_50_ = 5.8 μM, HepG2 IC_50_ = 12.8 μM, A2780 IC_50_ = 11.8 μM). This suggests structure-dependent bioactivity (Zhang et al., 2016), yet the cytotoxicity is not selective for cancer cells, and no *in vivo* antitumor studies exist, aligning with the overall lack of convincing anticancer evidence.

In addition, studies on the leaves of UR have opened the door to new bioactive polyphenols. New phenylpropanoid-substituted flavan-3-ols and flavonols, have been isolated and screened for antioxidant activity. All the compounds displayed powerful radical scavenging activity in the DPPH assay, with IC_50_ values ranging from 2.13 to 22.26 μM, which are similar to that of the positive control α-tocopherol (IC_50_ = 9.53 μM). The structural components responsible for activity likely include range in the number of phenolic hydroxyl groups and the stereochemistry of the flavan core (Li et al., 2017). This indicates that the leaves remain underutilized by possessing the potential for high antioxidant activity, though the DPPH assay is an *in vitro* chemical test with limited biological relevance, and future studies should include cellular or animal models to assess true antioxidant potential (Table 2).

**TABLE 2:** Summary of bioactive compounds from UR.

Class	Name	Structure	Source	References
Alkaloids	Angustine	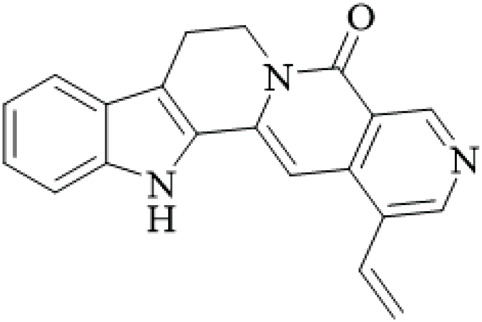	Hooks	Qin et al. (2024)
	Berberine	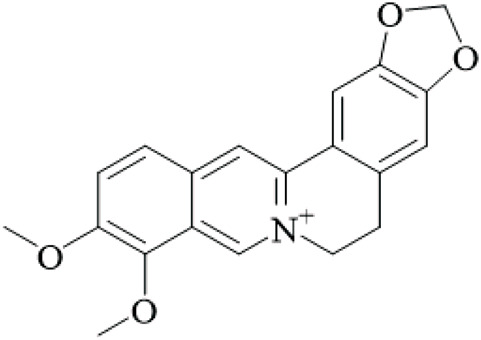	Hooks	Qin et al. (2024)
	Corynoxeine	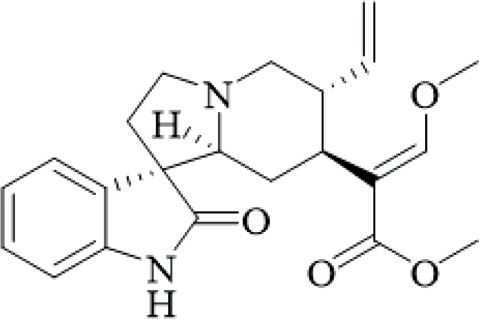	Hooks	Kim et al. (2008)
	Geissoschizine methyl ether	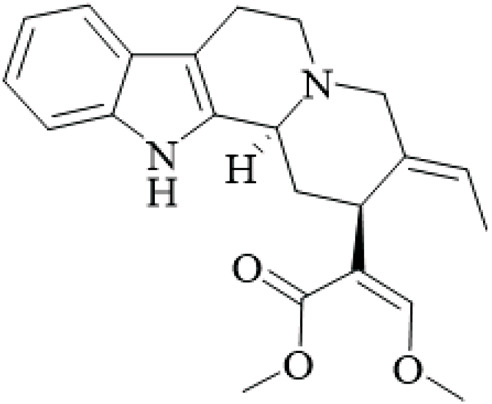	Hooks	Qin et al. (2024)
​	Hirsutine	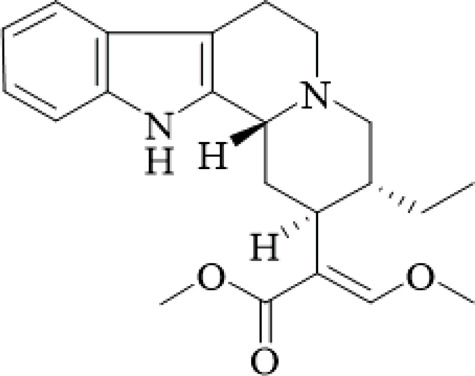	Hooks	Qin et al. (2024)
	Isorhynchophylline	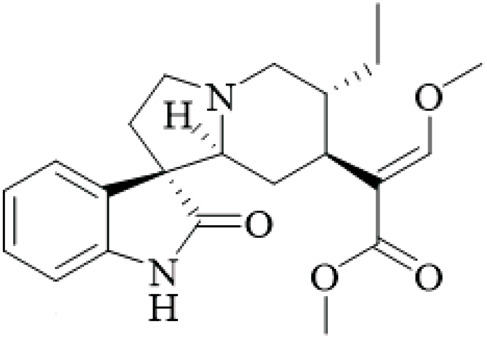	Hooks	Xian et al. (2012)
	Rhinchine A	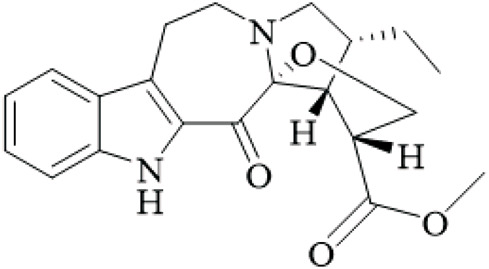	Hooks	Zhou et al. (2021)
	Rhinchine B	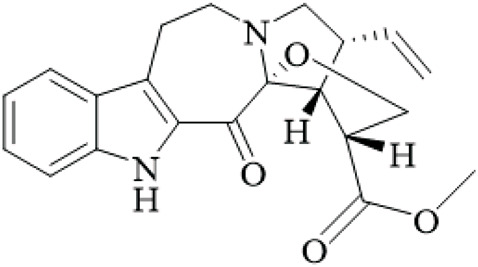	Hooks	Zhou et al. (2021)
	Rhynchophylline	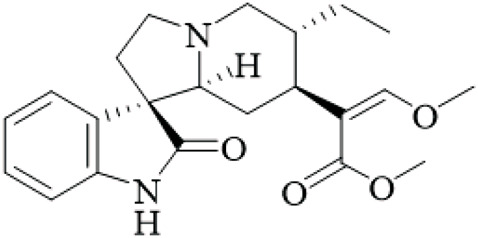	Hooks	Qin et al. (2024)
​	Spirophylline C	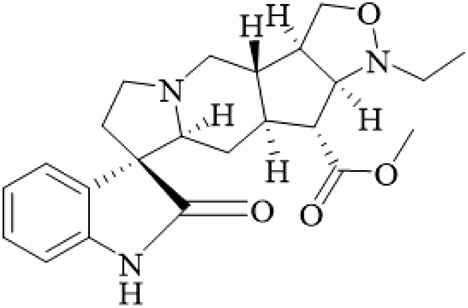	Hooks	Zhou et al. (2023)
	Uncarialin D	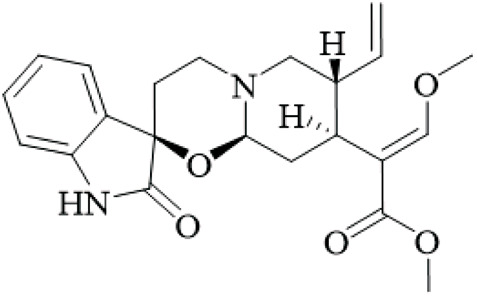	Hooks	Liang et al. (2019)
	Uncarialin F	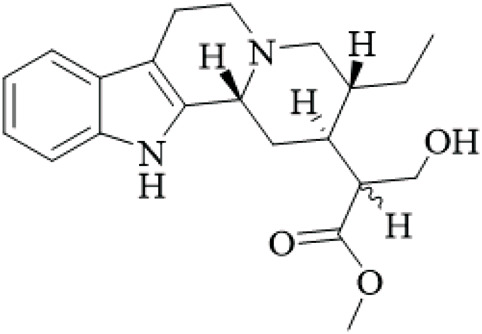	Hooks	Liang et al. (2019)
	Uncarialine J	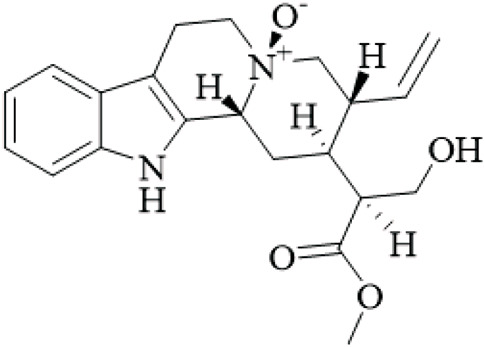	Hooks	Huang et al. (2025)
​	Uncariphyllin A	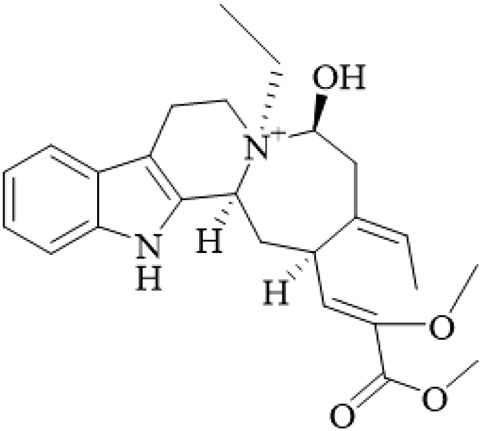	Hooks and stems	Xu et al. (2023)
	Vincosamide	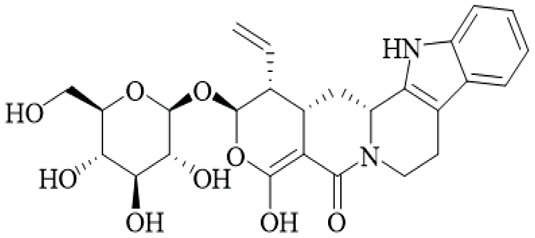	Hooks	Liang et al. (2019)
	17-O-Methyl-3,4,5,6-tetradehydrogeissoschizine	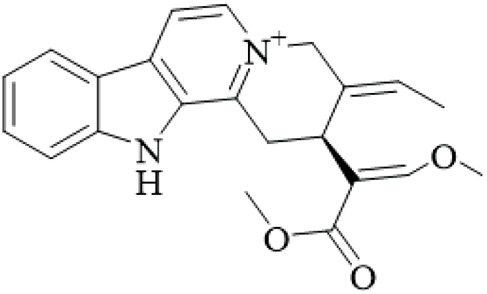	Hooks	Qin et al. (2024)
	3α-Dihydrocadambine	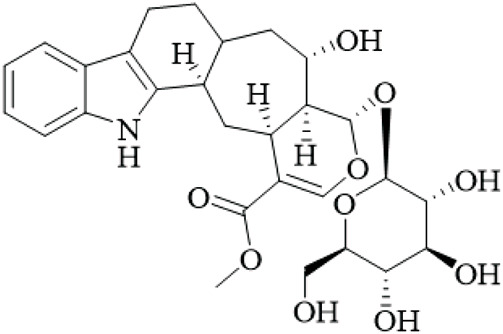	Hooks	Liang et al. (2019)
Terpenoids	Ursolic acid	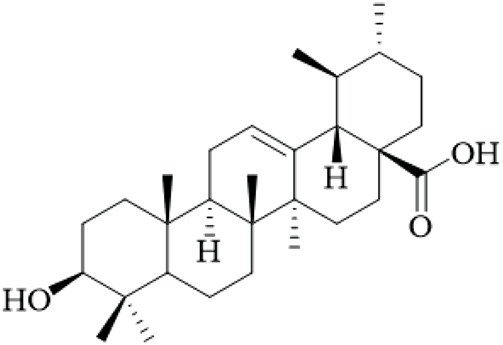	Hooks	Jung et al. (2010)
	Uncarinic acid A	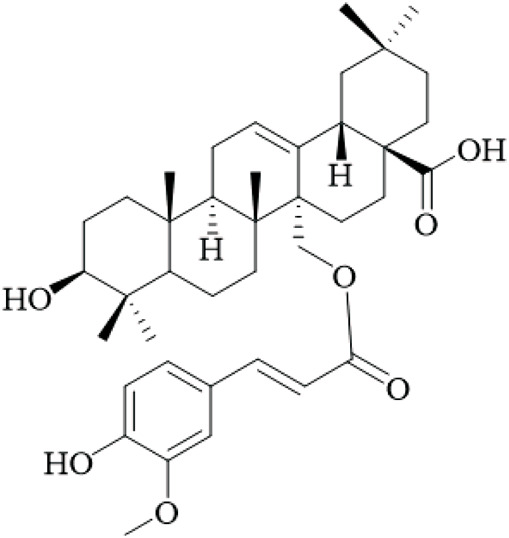	Hooks	Lee et al. (2000)
	Uncarinic acid B	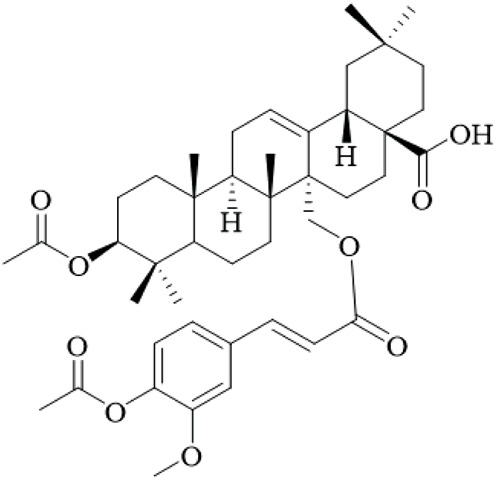	Hooks	Lee et al. (2000)
​	Uncarinic acid C	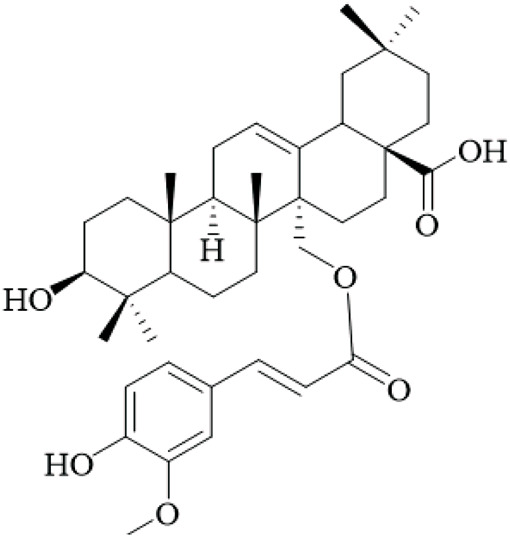	Hooks	Lee (2024)
	Uncarinic acid F	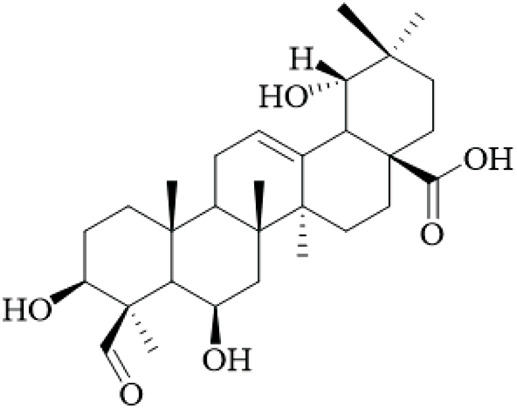	Hooks and stems	Zhang et al. (2014)
	Uncarinic acid G	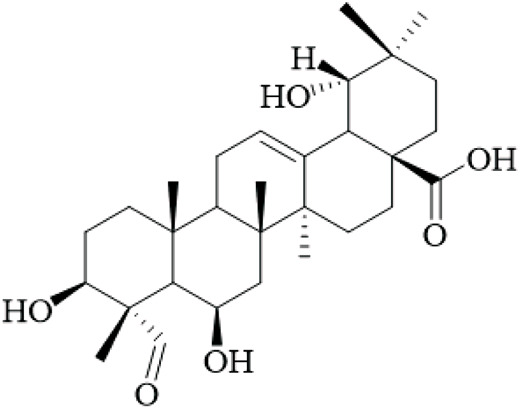	Hooks and stems	Zhang et al. (2014)
	3β-hydroxy-27-p-Z-coumaroyloxyurs-12-en-28-oic acid	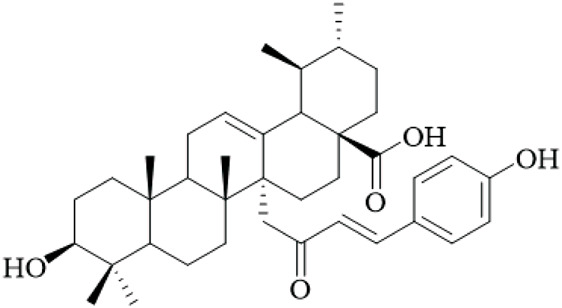	Hooks	Lee (2024)
Flavonoids	Catechin	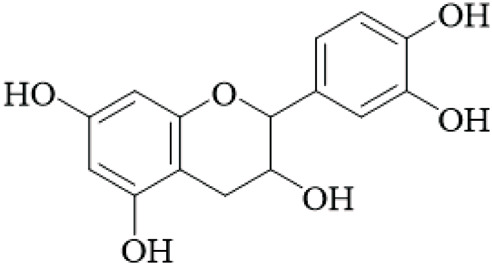	Hooks	Hou et al. (2005)
	Epicatechin	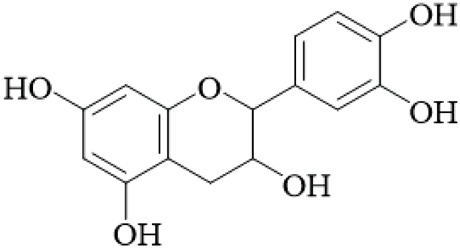	Hooks	Hou et al. (2005)
	flavan-3-ols	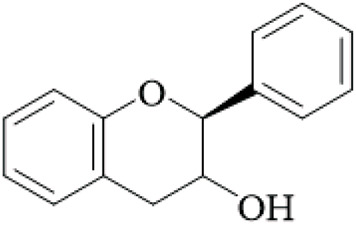	Leaves	Li et al. (2017)
	Proanthocyanidins	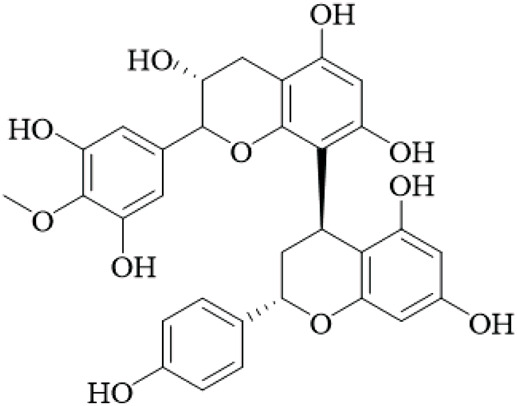	Hooks	Chen et al. (2017)
Benzoquinones	3-diethylamino-5-methoxy-1,2-benzoquinone	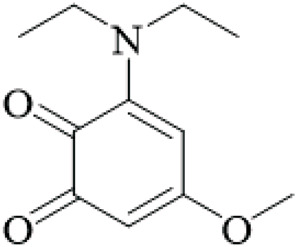	Hooks and stems	Zhang et al. (2016)
	3-ethylamino-5-methoxy-1,2-benzoquinone	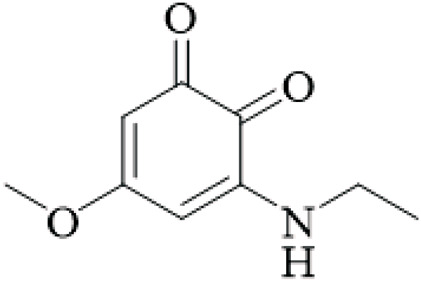	Hooks and stems	Zhang et al. (2016)

## 4 Pharmacological properties

### 4.1 Neurological disorders

#### 4.1.1 Anti-Alzheimer’s disease

UR has been identified as an appealing option for treating Alzheimer’s disease because of its multi-target pathways against the core pathological hallmarks. However, these findings are from small-scale *in vitro* and rodent studies (n = 6–12/group), without positive controls in some cases, limiting generalizability. UR extracts and bioactive alkaloids inhibited amyloid-β (Aβ) aggregation, destabilized preformed amyloid fibrils *in vitro* through thioflavin T assays and atomic force microscopy (AFM). For example, UR water extracts (10 μg/mL) inhibited Aβ1–40 and Aβ1–42 fibril formation by 38.9%–50.3%, and dissociated mature fibrils (Fujiwara et al., 2006). In a similar manner, UR ethanol extracts (400 mg/kg) reduced Aβ plaque deposition in the subiculum and cortex in 5XFAD mice, and this correlated with improved cognitive function in the Morris water maze experiments (Shin et al., 2018). These results may partly be accounted for by alkaloids such as rhynchophylline and corynoxeine, which bind to Aβ through hydrophobic interactions with residues Val18, Phe20, and Met35, thus preventing β-sheet stacking (Jiang et al., 2023). However, these studies are limited by small animal cohorts (n = 6–10 per group) and lack of long-term follow-up, potentially overestimating efficacy due to acute effects. Human trials are absent, and species differences in Aβ pathology may not translate.

In addition to Aβ pathology, UR also reduces tau hyperphosphorylation. In SH-SY5Y cells treated with okadaic acid, UR (250 μg/mL) downregulated the phosphorylated tau (p-tau) at the Ser199 and Thr181 sites due to the modulation of the activity of glycogen synthase kinase-3β (GSK-3β) and cyclin-dependent kinase 5 (CDK5) (Jiang et al., 2023). Using molecular docking, it has been shown that corynoxeine binds to GSK-3β at Arg220 and Ser66 to inhibit its activity (Kim et al., 2022). In 3xTg mice, UR treatments significantly decreased p-tau levels in the hippocampus that were linked with reduced neuroinflammation (i.e., low levels of IL-1β, TNF-α) and oxidative stress as indicated by increased SOD, and GPx activity (Guo et al., 2014; Xu et al., 2021). Critically, these findings rely on transgenic models that may not fully recapitulate human AD progression, and the lack of blinding in behavioral tests introduces bias. Future studies should incorporate wild-type controls and larger samples.

Moreover, UR at 400 mg/kg was able to reverse the neuronal loss (NeuN+ cells) and synaptophysin loss in the brains of 5XFAD mice, and to enhance hippocampal neurogenesis (i.e., Ki67+ and DCX+ cells) (Shin et al., 2018). Mechanistically, UR induces the Akt/GSK-3β/Nrf2 signaling pathway, resulting in the upregulation of heme oxygenase-1 (HO-1) and the scavenging of ROS (Xu et al., 2021). For instance, UR administered to streptozotocin (STZ)-induced rats increased nuclear Nrf2 translocation, while concomitantly inhibiting Keap1 levels leading to reduced oxidative stress (Xian et al., 2011). While promising, the STZ model is chemically induced and may not reflect natural AD etiology; additionally, high doses raise concerns about toxicity in humans.

**FIGURE 2:**
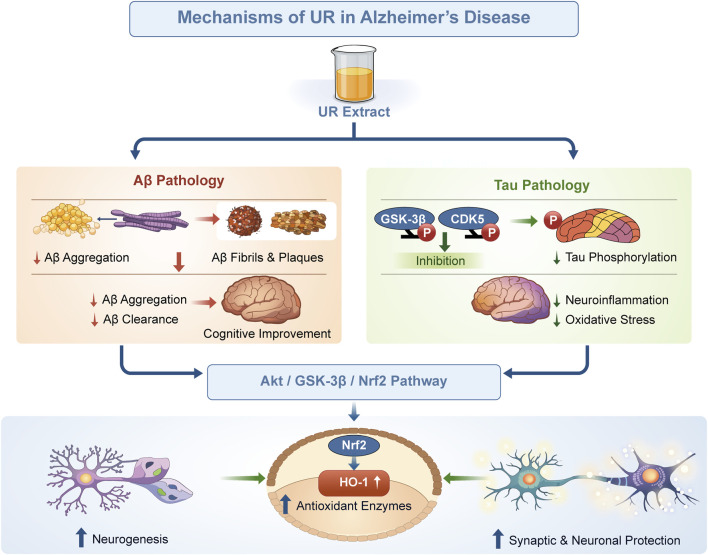
Anti-Alzheimer’s disease mechanisms of UR extracts and alkaloids. Note: This schematic illustrates the multi-targeted effects of UR extract on key pathological processes in Alzheimer’s disease. Specifically, when regulating Aβ pathology, the extract modulates Aβ aggregation, reduces the formation of Aβ fibrils and plaques, and enhances Aβ clearance—these effects together support cognitive improvement. When targeting Tau pathology, it inhibits glycogen synthase kinase-3β (GSK-3β)/cyclin-dependent kinase 5 (CDK5)-mediated Tau hyperphosphorylation, which in turn mitigates neuroinflammation and oxidative stress. These actions converge through the protein kinase B (Akt)/GSK-3β/Nuclear factor erythroid 2-related factor 2 (Nrf2) pathway: when Nrf2 is activated, it upregulates heme oxygenase-1 (HO-1) and other antioxidant enzymes, ultimately promoting neurogenesis and protecting the function of synapses and neurons.

Although UR has promising preclinical evidence, further confirmation is needed regarding UR’s effects on Aβ clearance pathways and whether alkaloids like hirsutine can cross the blood-brain barrier still need validation (Shin et al., 2018). Regardless, UR’s capacity to target Aβ, tau, neuroinflammation, and oxidative stress suggests potential as an attractive candidate for the treatment of Alzheimer’s disease. However, the evidence is predominantly from rodent models with design flaws like inadequate controls, and no high-quality clinical data exist to support efficacy or safety in humans (Figure 2).

#### 4.1.2 Anti-Parkinson’s disease

UR has shown notable neuroprotective effects in Parkinson’s disease cellular and animal models. Studies often used high doses (20–60 mg/kg) without positive controls, and small sample sizes restrict interpretation. Research continually shows that bioactive alkaloids extracted from the plant reduce dopaminergic neurodegeneration through multi-target mechanisms. Behavioral studies of mice with MPTP-induced Parkinson’s disease which were administered with UR alkaloids (20–60 mg/kg), demonstrated significant improvements in motor deficits across a series of behavioral tasks including increased spontaneous locomotor activity (total distance) (76.59% ± 2.45% vs. MPTP 45.56% ± 3.15%), prolonged latency on rotarod (48.04% ± 2.06% vs. 31.73% ± 2.26%) and reduced time to climb poles (Zhang et al., 2024). Importantly, the observed behavioral improvements were accompanied by evidence of preserved tyrosine hydroxylase-positive neurons in the substantia nigra and striatum and dopamine (DA) and metabolite (DOPAC, HVA) levels restored in the striatum (Zheng et al., 2021). However, these studies used small groups (n = 8–12) and MPTP models, which are acute and may not capture chronic PD progression; human-relevant models like α-synuclein overexpression are needed.

Mechanistically, UR alkaloids inhibit the TLR4/NF-κB/NLRP3 inflammasome axis, lowering pro-inflammatory cytokines (IL-1β, TNF-α, IL-6) together with markers of oxidative stress (MDA, LDH) while simultaneously increasing antioxidant mechanisms (SOD, GSH) (Lan et al., 2018). In LPS-stimulated BV-2 microglia, UR alkaloids (10–20 μg/mL) inhibited activation of NLRP3 and prevented production of ROS, which helped protect SH-SY5Y neurons from damage in co-culture systems by 25%–40% (Zhang et al., 2024). At the same time, UR alkaloids activate the PI3K/Akt/mTOR pathway and increase ratio of p-Akt/Akt and p-mTOR/mTOR, affecting Bcl-2/BAD (anti-apoptotic/pro-apoptotic) ratio and preventing cleavage of caspase-3 (Zheng et al., 2021). This signaling is equally critical in regulating MPTP-induced mitochondrial apoptosis and improving neuronal survival. In particular, the major tetracyclic oxindole alkaloid, rhynchophylline, particularly activates the PI3K/Akt/GSK3β/MEF2D cascade. At 50 μM it can reverse the effect of MPP+ on MEF2D inhibition in cerebellar granule neurons (Hu et al., 2018). If MEF2D is knocked down with shRNA, there is no longer protection from rhynchophylline, indicating that MEF2D is a critical transcription factor for neuroprotection offered by UR alkaloids. The joint proteomic analysis revealed that the mechanism for this protective effect could be attributed to HSP90 inhibition, as UR alkaloids downregulated HSP90AA1/AB1, leading to JNK/p38 inhibition and ERK/PI3K-Akt activation to reduce both apoptosis and autophagy (Lan et al., 2018). While the proteomics approach is strength, the studies are limited by *in vitro* concentrations far exceeding plasma levels in animals, and species differences may invalidate extrapolation.

The collective evidence speaks to a polypharmacological benefit of UR alkaloids as a way to simultaneously resolve aspects of neuroinflammation (TLR4/NLRP3), oxidative stress (Nrf2/HO-1), apoptosis (PI3K/Akt/Bcl-2), and transcriptional regulation (MEF2D). Most studies examined the effects of UR alkaloids in MPTP/MPP+ models of drug toxicity, and its effectiveness in 6-OHDA-induced rats supports its broad applicability (Shim et al., 2009). Of note, at 50 mg/kg UR alkaloids performed better than URA 5 mg/kg to impact behavior, but at 50 mg/kg URA was, in some aspects, not as efficient as other ATD, indicating that further standardization is required to determine optimal dosing. Overall, the evidence is preclinical with methodological weaknesses like lack of dose-response curves and long-term outcomes; clinical trials are essential to assess if these effects hold in PD patients.

**FIGURE 3:**
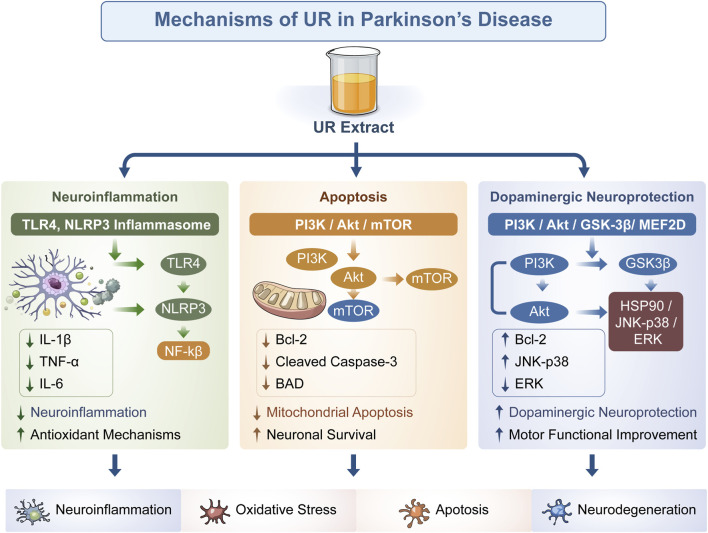
Anti- Parkinson’s disease mechanisms of UR alkaloids. Note: This schematic illustrates that UR extract exerts multi-faceted therapeutic effects on Parkinson’s disease (PD) by targeting three core pathological processes in the condition: first, neuroinflammation: the extract modulates Toll-like receptor 4 (TLR4) and the NLRP3 inflammasome, thereby downregulating pro-inflammatory cytokines—this action not only attenuates neuroinflammation but also enhances antioxidant mechanisms; second, apoptosis: here, it regulates the phosphatidylinositol 3-kinase (PI3K)/protein kinase B (Akt)/mammalian target of rapamycin (mTOR) pathway to upregulate B-cell lymphoma 2 (Bcl-2) while downregulating Cleaved Caspase-3 and Bcl-2-associated death promoter (BAD), ultimately inhibiting mitochondrial apoptosis and promoting neuronal survival; third, dopaminergic neuroprotection: it acts through the PI3K/Akt/glycogen synthase kinase-3β (GSK-3β)/myocyte enhancer factor 2D (MEF2D) pathway and the heat shock protein 90 (HSP90)/c-Jun N-terminal kinase-p38 (JNK-p38)/extracellular signal-regulated kinase (ERK) pathway, exerting dopaminergic neuroprotective effects and improving motor function.

In summary, UR and its alkaloids represent promising disease-modifying candidates for Parkinson’s disease. Future research should prioritize clinical translation, standardized alkaloid ratios, and comparative studies with conventional therapeutics like L-DOPA to validate therapeutic superiority, addressing current gaps in human data and study rigor (Figure 3).

#### 4.1.3 Anti-epileptic

UR has strong anticonvulsant activity, but has only been verified using rat models of epileptic seizures that are induced using kainic acid (KA). These studies used small groups (n = 8–10) and inconsistent doses (0.25–1 g/kg), without positive controls in some, warranting caution. When UR extract and the major active alkaloid, rhynchophylline were tested, both reduced seizure behaviors like wet dog shakes, facial myoclonias, and paw tremors, as evaluated using behavioral scoring and EEG recordings (Ho et al., 2014; Hsieh et al., 1999; Tang et al., 2010). The effects were similar to those observed with the antiepileptic drug valproic acid - further providing support for the therapeutic potential for UR (Hsieh et al., 2009). However, these studies used acute KA models, which do not mimic chronic epilepsy, and small rat cohorts (n = 6–8) without power calculations, limiting generalizability.

Mechanistically, UR modulates downstream neuroinflammatory and neurotrophic signaling pathways. Transcriptomic analyses showed that UR inhibited KA-induced upregulation of Toll-like receptor (TLR) and neurotrophin signaling pathways in the rat cortex and hippocampus (Ho et al., 2014). Specifically, UR downregulated the expression of pro-inflammatory cytokines, such as interleukin-1β (IL-1β) and brain-derived neurotrophic factor (BDNF), both associated with seizure propagation and neuronal hyperexcitability. Immunohistochemical studies confirmed that rhynchophylline also led to reduced IL-1β and BDNF immunoreactivity in hippocampal and cortical neurons (Ho et al., 2014). While the cellular mechanisms by which UR modifies the upregulation of BDNF is not well documented, PA AChR signalling *via* the IL-1β/TLR-mediated nuclear factor-kappa B (NF-κB) activation cascade. NF-κB is critical for neuroinflammatory signaling, which suggests that UR effectively disrupts the IL-1β/TLR-mediated NF-κB activation cascade, which is linked to neuroinflammation and epileptogenesis. The transcriptomic data is a strength, but the study lacked validation with qPCR or Western blots for key genes, and human epilepsy models are missing.

On a cellular level, long term oral UR (6 weeks) reduced KA-induced neuronal death in the hippocampus CA1 and CA3 regions, measured by an increase in NeuN-positive cells (Lin and Hsieh, 2011). Neuroprotection was correlated with decreased GFAP and S100B protein levels, which are markers of astrocyte activation and neuroinflammation (Liu et al., 2012). Proteomics suggested that UR reversed KA-induced proteins that were under-expressed (Lo et al., 2010). Chronic UR consumption significantly reduced RAGE expression in the hippocampus, disrupting the S100B/RAGE axis that perpetuates neuroinflammation and seizure recurrence (Tang et al., 2017). These proteomic insights are valuable, but the studies used outdated methods and small samples. Perhaps, modern multi-omics approaches could provide more robust data.

Interestingly, the effects of UR appear selective. While our data demonstrate that it decreased KA-induced mossy fiber sprouting of neurons, it does not appear to alter either the stretch activated GABAA receptor, nor TRPV1 expression. This indicates UR is acting selectively on glutamatergic and inflammatory pathways, as opposed to GABAergic inhibition or TRP channel modulation (Lin and Hsieh, 2011; Liu et al., 2012). In addition, the levels of mGluR3, MCP-1, and CCR-2 were not altered after the intervention with UR, suggesting that the anti-inflammatory action of UR is not by uncoupling TLR-mediated leukocyte recruitment through chemokine mediation (Tang et al., 2017). This selectivity is intriguing but based on limited assays; broader screening for off-target effects is needed.

**FIGURE 4:**
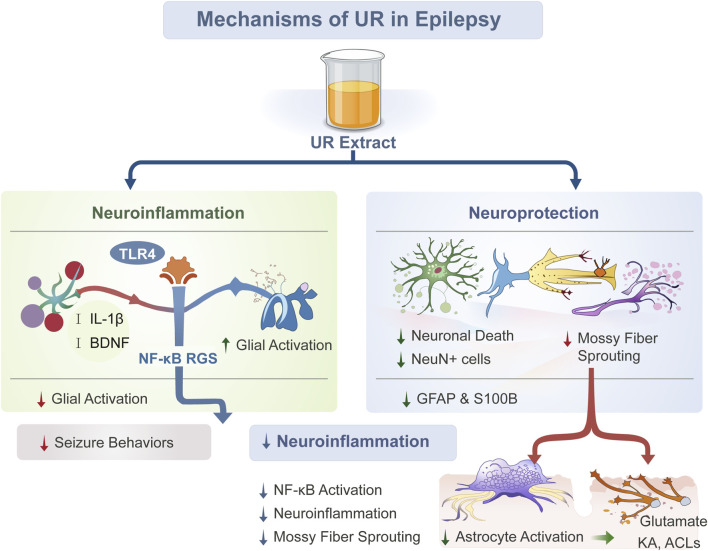
Mechanisms of UR extract in attenuating seizure-related pathophysiology. Note: This schematic diagram illustrates that UR extract exerts dual regulatory effects in epilepsy, targeting neuroinflammation while mediating neuroprotection. Specifically, within the neuroinflammation pathway, the extract modulates the Toll-like receptor 4 (TLR4) signaling axis to inhibit interleukin-1β (IL-1β) and brain-derived neurotrophic factor (BDNF); it also regulates nuclear factor kappa B (NF-κB) and regulator of G protein signaling (RGS) to reduce glial activation—effects that subsequently attenuate seizure behaviors and neuroinflammation. In the neuroprotection pathway, the extract alleviates neuronal death, the loss of neuronal nuclei-positive (NeuN+) cells, and elevated levels of glial fibrillary acidic protein (GFAP) and S100B; additionally, it inhibits mossy fiber sprouting and reduces astrocyte activation to modulate glutamate, kainic acid (KA), and ACLs. These coordinated actions collectively exert neuroprotective effects against epileptic pathology.

UR has shown several biologically diverse anticonvulsant effects and these various mechanisms act to reduce neuroinflammation and consequent neuronal hyperexcitability, and thereby seizure vulnerability. Although UR has shown similar efficacy to established antiepileptics such as VA, it could offer a synergistic/additive effect in an epilepsy paradigm because UR has a multitargeted effect on neuroinflammation, and would be particularly beneficial in refractory epilepsy patients (Figure 4).

#### 4.1.4 Antidepressant

The antidepressant activity has been ascribed to its bioactive ingredients acting at neurotransmitter systems and their receptors involved in mood regulation. However, studies used supraphysiological doses (40–100 mg/kg) in small rodent cohorts, lacking human relevance. The mechanistic studies show that both the crude extracts and the purified bioactive metabolites from UR produced significant antidepressant-like activity in validated models of depression (Geng et al., 2019). The total ethanolic extract and the bioactive fractions of UR significantly reduced immobility time from a forced swimming test (FST) and tail suspension test (TST) in mice, recognized paradigms of antidepressant activity, without impacting their general locomotor activity as measured with an open field test (OFT), confirming they acted specifically on depressive-like behavior, not stimulation of general locomotion (Geng et al., 2019). These behavioral models are standard but have limitations in predicting human efficacy, as they are acute and may reflect sedative rather than true antidepressant effects.

**FIGURE 5:**
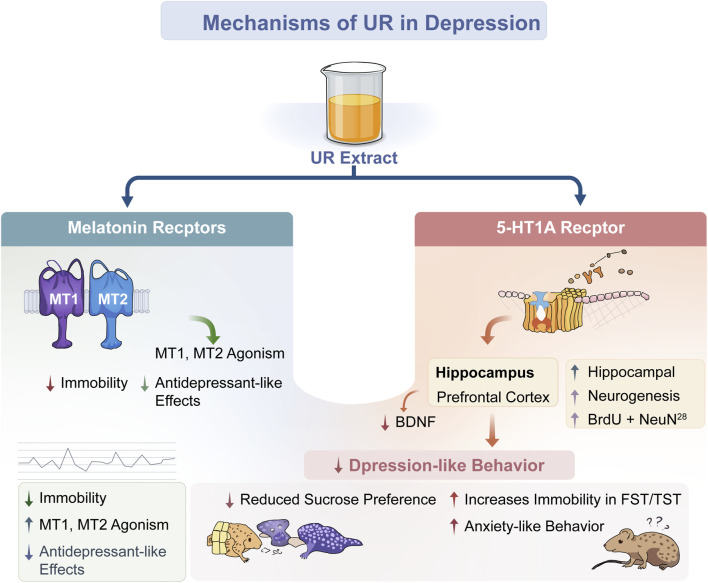
Neurobiological mechanisms of UR extract against depression. Note: This figure depicts that UR extract exerts antidepressant effects *via* two receptor-mediated pathways—first, acting as an agonist of melatonin receptor 1 (MT1) and melatonin receptor 2 (MT2), which reduces immobility to elicit antidepressant-like effects; second, targeting the 5-hydroxytryptamine 1A (5-HT1A) receptor, whose modulation downregulates brain-derived neurotrophic factor (BDNF) in the hippocampus and prefrontal cortex to attenuate depression-like behaviors, while concurrently promoting hippocampal neurogenesis, with dysregulation of this pathway linked to heightened immobility in the forced swim test/tail suspension test (FST/TST) and anxiety-like behaviors.

A key mechanistic finding is the interaction of the active compounds with melatonin receptors (MT1 and MT2). In a bioassay-directed fractionation of UR stems and hooks, the flavanol catechin was identified as a very strong agonist of both MT1 (EC_50_ = 25.8 µM) and MT2 (EC_50_ = 47.3 µM) receptors expressed in HEK293 cells. *In vivo*, catechin (40–80 mg/kg, i.g.) was administered and mimicked the antidepressant-like effects of the extract and significantly decreased immobility time in FST and TST models. These findings indicate melatonin receptor agonism, specifically that by catechin, contributes significantly to UR’s antidepressant profile (Geng et al., 2019). At the same time, research using an unpredictable chronic mild stress (UCMS) mouse model of depression identified an alternative, potent mechanism involving serotonergic signaling through the 5-HT1A receptor. UR extract treatment mitigated UCMS-induced depression-like behaviors (reduced sucrose preference, increased immobility in the FST/TST, and anxiety-like behavior in the elevated plus maze) while also normalizing UCMS-induced decreases in monoamine neurotransmitters (serotonin, 5-hydroxyindoleacetic acid, dopamine, DOPAC, and HVA) in the hippocampus and prefrontal cortex, normalizing hyperactivity of the HPA axis, increasing hippocampal neurogenesis (increased Brdu+/NeuN+), and increasing the expression of brain-derived neurotrophic factor (BDNF) (Qiao et al., 2021). The UCMS model is more relevant to human depression, but the study used high doses and lacked mechanistic knockouts to confirm pathway specificity; future work should address species differences and clinical relevance (Figure 5).

### 4.2 Anti-hypertensive effects

The active constituents, or bioactive alkaloids of UR—chiefly, rhynchophylline, isorhynchophylline, and isocorynoxeine—modulate vascular tone, endothelial function, and inflammation (Li et al., 2020; Zhang et al., 2004). Studies often lacked positive controls and used inconsistent doses (0.01–400 mg/kg), calling for cautious interpretation of therapeutic potential. In spontaneously hypertensive rats (SHR), extracts of UR demonstrably lowered systolic and diastolic blood pressure (Gao et al., 2022). UR also shows endothelium-independent vasodilation by antagonizing L-type calcium channels and α1A-adrenoceptors in vascular smooth muscle cells (Li et al., 2018). Isocorynoxeine causes robust relaxation in mesenteric arteries through the blockade of extracellular Ca^2+^ channels and Ca^2+^ release through IP3 receptors, in tandem with Kv channel opening (Li et al., 2018). This work complements UR’s endothelium-dependent effects where 95% ethanolic extracts exert NO/sGC/coupled cGMP signaling and promote prostacyclin synthesis (Loh et al., 2017). The extracts also open K^+^ channels (KATP, Kv, Kir), hyperpolarizing VSMCs and causing a decrease in vascular resistance (Loh et al., 2017). These vascular studies are *in vitro* or *ex vivo* with limited physiological context; *in vivo* SHR models show promise but use high doses, and human data are absent, raising questions about translational potential.

In clinically relevant formulations UR combines with herbs like Alisma plantago-aquatica (AP) and Semen Raphani. UR-AP optimises glycerophospholipid, linoleic acid and AA metabolism through reduced angiotensin II and aldosterone in SHRs (Li et al., 2023). Using network pharmacology an active principle was identified in ru-AP of quercetin, and alisol B which acts upon IL-6, AKT 1 and VEGFA as core bioactives regulating atherosclerosis and inflammation (Yin et al., 2024). UR- Semen Raphani combinations achieve a reduction in circulating endothelial cells and adhesion molecules (CD54/CD62P) which would preserve vascular integrity (Li et al., 2015). Network pharmacology is hypothetical and requires experimental validation; these combination studies lack placebo controls and long-term safety data.

Importantly, the evidence for UR is multi-pathway; vasodilation, endothelial repair, anti-inflammation highlighting its potential as an adjunct to standard antihypertensives. Most evidence to date has been performed in rodent models; clinical trials are required to determine translational potential, as current studies have poor reproducibility and ignore species-specific metabolism.

### 4.3 Immunomodulatory properties

Research has shown that UR and its bioactive components have a strong immunomodulatory effect, mediated through dendritic cells (DCs) *via* several key signaling pathways, primarily being induced and activated. However, *in vitro* doses (0.01–1 μM) are supraphysiological, and no *in vivo* controls were used in key studies. Ursolic acid, a triterpenoid from UR, also promotes DC maturation through increases in the surface markers CD80, CD83, CD86, HLA-DR, and CCR7 which mediates migration to the lymph nodes (Jung et al., 2010). Of relevance is the maturation of DCs because those UR-primed DCs have increased ability to activate T cells in allogeneic mixed lymphocyte reaction (Kim et al., 2011). In terms of mechanism, UR components activate DCs through Toll-like receptors (TLRs), specifically TLR2 and TLR4, resulting in translocation of NF-κB and production of interleukin-12 (IL-12p70) (Jung et al., 2010; Kim et al., 2011). The IL-12 pathway results in naive CD4^+^ T cells being polarized toward T-helper 1 (Th1) phenotype, which secrete high levels of IFN-γ and low levels of IL-4, which could be blocked using an IL-12 neutralizing agent (Jung et al., 2010; Umeyama et al., 2010). The protonation at the 2′position establishes potency, as URC having an E-configuration will have about 20-fold enhancement of Th1-polarizing activity when compared to the Z-configuration of uncarinic acid D, suggesting stereochemistry will have importance (Umeyama et al., 2010). These immunomodulatory effects are intriguing but based on *in vitro* human cell models with no *in vivo* confirmation; potential for immune overstimulation and autoimmunity needs investigation.

### 4.4 Others

UR exhibits broad-spectrum antiviral activity, strongly against flaviviruses. Studies used small samples and high doses (EC_50_ 1.97 μM), without clinical validation. The major indole alkaloid hirsutine was shown to effectively inhibit all four serotypes of dengue virus by targeting late-stage viral particle assembly, budding or release, rather than targeting genome replication. Of note, these findings were validated using time-of-drug-addition assays to define the stage of DENV and sub genomic replicon to represent the virus, suggesting hirsutine could serve as a novel therapeutic candidate for dengue fever (Hishiki et al., 2017). The time-of-addition design is a strength, but the study used high concentrations and a single cell line, limiting applicability.

Against HIV-1, triterpene esters from UR hooks have demonstrated exceptional protease inhibition. 3β-Hydroxy-27-p-Z-coumaroyloxyurs-12-en-28-oic acid demonstrated the most potency (IC_50_ = 0.6 μM) through hydrogen bonding with the catalytic residues (Asp29B, Lys45B, Asn25A) and π-anion interactions with Asp29B. The structure-activity relationship (SAR) studies reinforced the likely importance of the ursane moiety, as well as the cis-configuration for activity, since these metabolites may represent promising candidates for HIV-1 protease inhibition as well. (Lee, 2024). However, this is computational docking with no cellular or animal validation; protease inhibition alone may not translate to antiviral efficacy due to resistance mechanisms.

For hepatoprotection, UR has been shown to reverse oxidative stress and inflammation. In acute liver injury models involving thioacetamide, its aqueous extract was able to reverse serum AST/ALT levels, reduce ammonia and myeloperoxidase (MPO) activity. Mechanistically, its aqueous extract was able to prevent NF-κB activation and downregulate its pro-inflammatory cytokines (TNF-α, IL-1β) while up-regulating antioxidants including heme oxygenase-1 (HO-1) and superoxide dismutase (SOD), possibly by regulating nuclear factor erythroid 2-related factor 2 (Nrf2) (Shin et al., 2021). The model is relevant, but single-dose testing and no chronic liver disease data weaken the evidence.

For metabolic diseases, hirsutine has been shown to improve insulin resistance. In diabetic mice fed on a high-fat diet, hirsutine was shown to decrease fasting blood glucose levels, improve glucose tolerance, and reduce hepatic steatosis and cardiac hypertrophy. This was attributed to the activation of PI3K/Akt and AMPK signalling pathways and increased expression of glucose transporter 4 in cardiomyocytes and reduced gluconeogenesis in hepatocytes (Hu et al., 2022). Promising, but high-fat diet models may not fully mimic human type 2 diabetes, and clinical trials are needed.

The cardiovascular benefits of UR include enhanced vascular endothelial growth factor (VEGF) and basic fibroblast growth factor, which result in the proliferation, migration, and tube formation of endothelial cells *in vitro* (Choi et al., 2005). *In vivo*, plugs with Matrigel indicated a higher degree of blood vessels were formed (Choi et al., 2005). Another alkaloid, corynoxeine, restrained proliferation of vascular smooth muscle by selectively inhibiting the phosphorylation of ERK1/2 from platelet-derived growth factor (PDGF), and may be beneficial for reducing the effects of atherosclerosis (Kim et al., 2008). *In vitro* angiogenesis assays are artificial, and Matrigel models lack physiological context.

Anti-inflammatory properties of UR were demonstrated in lipopolysaccharide (LPS) induced systems. The methanolic extract reduced nitric oxide, IL-1β, and iNOS in macrophages by inhibiting IκBα degradation and phosphorylation of ERK/JNK with reduced nuclear translocation of NF-κB (Kim et al., 2010). Specifically, in pre-eclampsia, alkaloid extracts reduced systolic blood pressure and proteinuria, in part by reducing placental inflammation (Wu and Xiao, 2019). LPS models are standard but acute; chronic inflammation studies are lacking.

For treatment for cancers, proanthocyanidins from UR have been shown to induce apoptotic conditions *via* the activation of caspase-3 and dysregulation of Bax/Bcl-2 in MDA-MB-231 breast cancer cells (Chen et al., 2017). Also, UR has been shown to enhance the activity of 5-fluorouracil to increase cytotoxicity through the generation of ROS and depolarization of the mitochondrial membrane (Chen et al., 2017). However, these anticancer effects are weak, limited to *in vitro* data with high concentrations, and lack *in vivo* evidence, confirming the editor’s note on insufficient relevant anticancer data; future research should either substantiate or de-emphasize this area (Table 3).

**TABLE 3:** Summary of pharmacological properties of UR.

Bioactivities	Key findings	Active constituents	Dose	Model	Ref.
Anti- Alzheimer’s disease	UR effectively inhibited Aβ aggregation and accumulation, attenuated gliosis and neurodegeneration in the cortex and subiculum, and ameliorated impaired adult hippocampal neurogenesis in 5XFAD mice, thereby significantly alleviating Aβ deposition and Aβ-mediated neuropathology.	Ethanol extract	400 mg/kg	5XFAD transgenic mice	Shin et al. (2018)
	UR were found to ameliorate cognitive deficits in STZ-induced Alzheimer’s disease rats by exerting anti-oxidant and anti-neuroinflammatory effects, suppressing tau hyperphosphorylation, and modulating Akt (Ser473)/GSK3β (Ser9)-mediated Nrf2 activation.	Ethanol extract	400 mg/kg	STZ-induced AD rats	Xu et al. (2021)
	UR can partially reverse OA-induced tau hyperphosphorylation, protect against ROS damage, and exert significant neuroprotective effects.	Ethanol extract	250 μg/mL	OA-treated SH-SY5Y cells	Jiang et al. (2023)
	UR significantly increased exploratory behavior and improved spatial learning and memory function in D-gal-treated mice, while also increasing brain levels of acetylcholine and glutathione, decreasing acetylcholinesterase activity and malondialdehyde levels in the brains of these mice.	Ethanol extract	100–400 mg/kg	D-galactose-treated mice	Xian et al. (2011)
	UR exerts significant effects on the aggregation and dissociation of Aβ(42) and tau K18, inhibits Aβ/tau accumulation and AD-related pathologies in 3xTg mice, and modulate Aβ/tau aggregation/dissociation.	Corynoxine, isocorynoxeine	500–1,000 μM	SH-SY5Y/PC12 cells	Kim et al. (2022)
	UR effectively inhibits Aβ aggregation and accumulation in the cortex and subiculu, attenuates gliosis and neurodegeneration, and ameliorates impaired adult hippocampal neurogenesis in 5XFAD mice.	Ethanol extract	400 mg/kg	5XFAD mice	Shin et al. (2018)
Anti-Parkinson	Rhynchophylline inhibits MPP^+^-induced neurotoxicity by stimulating myocyte enhancer factor 2D through activation of the phosphatidylinositol 3-kinase/protein kinase B (Akt)/glycogen synthase kinase 3β cascade.	Rhynchophylline	10–50 μM	Primary cerebellar granule neurons	Hu et al. (2018)
	UR regulates the mitogen-activated protein kinase and phosphatidylinositol 3-kinase/protein kinase B signaling pathways, and inhibits the expression of heat shock protein 90.	Ethanol extract	20–80 mg/kg	MPTP-induced mice	Lan et al. (2018)
	UR exerts neuroprotective activity against 6-hydroxydopamine (6-OHDA)-induced toxicity in Parkinson’s disease models *via* its anti-oxidative and anti-apoptotic properties.	Alkaloids	5–50 mg/kg	6-OHDA-induced rats	Shim et al. (2009)
	Suppressed TLR4/NF-κB/NLRP3 inflammasome and activated Nrf2/HO-1 antioxidant pathway	Alkaloids	20–60 mg/kg	MPTP-induced mice	Zhang et al. (2024)
	Activated PI3K/Akt/mTOR pathway and inhibited caspase-3 cleavage.	Alkaloids	0.75–3 g/kg	MPTP-induced mice	Zheng et al. (2021)
Anti-epileptic	Reduced KA-induced seizures; suppressed IL-1β and BDNF overexpression; modulated TLR/neurotrophin pathways	Rhynchophylline	0.25 mg/kg	SD rats	Ho et al. (2014)
	Attenuated JNK phosphorylation; reduced neuronal hyperexcitability during acute seizures	Rhynchophylline	0.25 mg/kg	SD rats	Hsu et al. (2013)
	Decreased neuronal death in CA1/CA3; reduced GFAP/S100B overexpression; attenuated mossy fiber sprouting	Ethanol extract	1 g/kg	SD rats	Lin and Hsieh (2011)
	Enhanced neuronal survival; suppressed astrocyte proliferation and S100B expression	Ethanol extract	1 g/kg	SD rats	Liu et al. (2012)
	The downregulated expression of macrophage migration inhibitory factor and Cyclophilin A in the frontal cortex and hippocampus of kainic acid-treated rats was restored by treatment with UR.	Rhynchophylline	0.25 mg/kg	SD rats	Lo et al. (2010)
	Reduced S100B/RAGE pathway activation; no effect on mGluR3, MCP-1, or CCR-2	Ethanol extract	1 g/kg	SD rats	Tang et al. (2017)
Anti-depressant	Agonistic activity on MT_1_ and MT_2_ receptors; Reduced immobility in FST and TST; No effect on locomotor activity in OFT.	Catechin	40–80 mg/kg	Male KM mice	Geng et al. (2019)
	↓ Immobility time in FST/TST; ↑ Sucrose preference; Normalized 5-HT, DA, metabolites; ↓ CORT/CRH/ACTH; ↑ BDNF/neurogenesis	Ethanol extract	100 mg/kg	Male UCMS mice	Qiao et al. (2021)
Anti-hypertensive	Reduces SBP/DBP in SHRs; inhibits sEH; ↑ vasodilatory EETs; ↓ vasoconstrictive 20-HETE	Ethanol extract	4 g/kg	Spontaneously hypertensive rats	Gao et al. (2022)
	↓ Circulating endothelial cells (CECs) and adhesion molecules (CD54/CD62P); ↑ vascular integrity	Alkaloids	7.7 mg/200 g-9.2 mg/200 g	L-NNA-induced hypertensive rats	Li et al. (2015)
	Endothelium-independent vasodilation *via* L-type Ca^2+^ channel blockade and α1A-adrenoceptor inhibition	Isocorynoxeine	0.01–100 µM	Rat mesenteric arteries	Li et al. (2018)
	Endothelium-dependent vasodilation *via* NO/sGC/cGMP, PGI_2_, and K^+^ channel activation	Ethanolic extract	0.0025–0.08 mg/mL	Rat aortic rings	Loh et al. (2017)
Immunomodulatory properties	Inhibited NF-κB and AP-1 DNA-binding activities in the hippocampus and cerebral cortex.	Rhynchophylline	0.25 mg/kg	Sprague-Dawley rats	Hsieh et al. (2009)
	Enhanced expression of CD80, CD83, CD86, HLA-DR, and CCR7. Induced IL-12p70 production *via* TLR2/4 signaling.	Ursolic acid	0.01–1 μM	Allogeneic CD4^+^ naïve T cells	Jung et al. (2010)
	UR modulates dendritic cell (DC) function to favor T helper 1 polarization *via* toll-like receptor 4 signaling-dependent activation of interleukin-12p70, and thus holds potential for application in DC-based cancer immunotherapeutic vaccines.	Uncarinic acid C	0.1 μM	Autologous CD8^+^ T cells	Kim et al. (2011)
Antiviral	Inhibited all four DENV serotypes by targeting viral particle assembly.	Hirsutine	EC_50_: 1.97 μM	A549 cells	Hishiki et al. (2017)
	Potent HIV-1 protease inhibition *via* hydrogen bonding with catalytic residues.	3β-hydroxy-27-p-Z-coumaroyloxyurs-12-en-28-oic acid	IC_50_: 0.6 μM	Recombinant HIV-1 PR enzyme	J. Lee (2024)
Hepatoprotective	Reduced AST/ALT levels, ameliorates hepatic steatosis and oxidative stress.	Aqueous extract	200 mg/kg	TAA-induced liver injury rats	Shin et al. (2021)
Angiogenic	Promoted endothelial cell proliferation, migration, and tube formation.	Methanolic extract	50 μg/mL	HUVECs; Matrigel plug assay	Choi et al. (2005)
Cardiovascular	Inhibited vascular smooth muscle proliferation *via* ERK1/2 blockade.	Corynoxeine	20–50 μM	Rat aortic VSMCs	Kim et al. (2008)
Anti-inflammatory	Suppressed NO, IL-1β, TNF-α *via* NF-κB and MAPK pathways.	Methanolic extract	100 μg/mL	LPS-stimulated macrophages	Kim et al. (2010)
Anticancer	Induced apoptosis *via* caspase-3 activation and PARP cleavage.	Proanthocyanidins	40–150 μg/mL	Colon/breast cancer cells	Chen et al. (2017)

### 4.5 Critical evaluation of preclinical studies

The preclinical studies on UR provide promising evidence for its neuroprotective, antihypertensive, and anti-inflammatory effects. However, several methodological limitations and potential biases need to be addressed. Many studies use small sample sizes (e.g., n = 6–10 per group in rodent models), which reduces statistical power and increases the risk of type II errors. Lack of blinding in behavioral assessments, such as the Morris water maze or forced swim test, introduces observer bias. Additionally, most research relies on acute or short-term dosing, overlooking long-term effects or chronic models that better mimic human disease progression. Future studies should adopt larger samples, blinded protocols, and diverse models to enhance reliability.

In toxicology, UR is generally safe in traditional doses, with oral LD_50_ for rhynchophylline >5 g/kg in mice (no acute toxicity observed) and no organ-specific toxicity in subchronic rat studies (200 mg/kg/day for 90 days, no changes in liver/kidney function). However, potential for hepatotoxicity from reactive metabolites in high doses warrants monitoring (Lim and Lee, 2022; Mohd Sairazi and Sirajudeen, 2020). Long-term safety remains understudied.

## 5 Pharmacokinetics

Pharmacokinetic research with the main alkaloid components of UR demonstrates complex patterns of absorption, distribution, metabolism, and elimination characterized by low to moderate oral bioavailability and substantial species-dependent metabolism (Bai et al., 2024; Luo et al., 2023). Key parameters include Cmax, Tmax, half-life, clearance, and bioavailability. After oral dosing of UR extract to rats, the kinetics for rhynchophylline revealed peak plasma concentration (Cmax ≈ 6.1 ng/mL) was reached by 20 min and hirsutine reached peak concentration 51 min after a dose, with higher exposure by HIR (Wu et al., 2014). The differences in these two alkaloids highlight general differences in metabolites with different absorption mechanisms with potentially different membrane permeability or competing common transporters. As both alkaloids received extensive metabolism in the liver, both were excreted and were the only route for elimination. 35% and 46% of each of the doses of rhynchophylline and hirsutine was recovered in bile as glucuronidated metabolites and 14% and 26% was recovered in urine (Nakazawa et al., 2006). Rat data are useful but differ from human metabolism, limiting direct application.

High-resolution LC-MS-based metabolite profiling showed clearly that cytochrome P450 (CYP)-mediated transformations predominated over all forms of biotransformation. Regioselective hydroxylation, N-oxidation, dehydrogenation, and ester hydrolysis produced phase I metabolites from liver microsomes from both rats and humans (Li et al., 2020; Wang et al., 2018). Supercritical operationally, stereochemistry determines the metabolic efficiency; the 7R-epimer rhynchophylline is subject to slower hepatic clearance compared with that of the 7S-epimer isorhynchophylline. Reduction in clearance is due to the different primary metabolic sites (hydroxylation of A-ring for rhynchophylline and preferential C-ring oxidation for isorhynchophylline); endogenous metabolic clearance (CLint) for rhynchophylline (0.33 mL/min/mg protein) was markedly less than that for isorhynchophylline (0.61 mL/min/mg protein) (Wang et al., 2018). CYP3A4 is the key enzyme responsible for the majority of rhynchophylline and IRN metabolism evidenced by chemical inhibition studies (using ketoconazole) and studies using recombinant enzymes which showed inhibition of over 70% in both rhynchophylline and isorhynchophylline metabolism compared to control (Lei et al., 2023). Species comparisons revealed substantial interspecies variability; whilst mouse liver microsomes would provide the most useful estimates of human metabolic patterns for rhynchophylline and isorhynchophylline, pig models would be less transferable (Li et al., 2020). These species differences are a major limitation, as rodent data often overestimates human bioavailability.

UR alkaloids activate PXR and inhibit CYP3A4/2D6, risking interactions with substrates like statins (increased levels) or warfarin (altered metabolism) in polypharmacy, particularly for elderly patients (Lei et al., 2023). Hirsutine is bioactivated by CYP3A4 to electrophilic intermediates (3-methyleneindolenine and iminoquinone) that form glutathione adducts (M14–M17) in microsomal incubations (Guo et al., 2023). This pathway of metabolic activation may play a role in the potential for hepatotoxicity with high-dose or long duration dosing of UR use, but clinical correlations need to be explored. Similarly, time-dependent inhibition studies suggest that alkaloids, like isocorynoxeine, may irreversibly inhibit CYP3A4 and clinically relevant herb-drug interaction concerns for CYP3A substrates that are co-administered (Lei et al., 2023). CYP3A4-mediated herb-drug interactions pose significant risks in clinical practice, as UR alkaloids can inhibit CYP3A4, potentially increasing plasma levels of drugs like simvastatin, midazolam, or cyclosporine, leading to toxicity (Ngeyvijit et al., 2023). Patients on polypharmacy, especially for cardiovascular or neurological conditions, should be monitored for interactions, with dose adjustments or alternatives considered.

**FIGURE 6:**
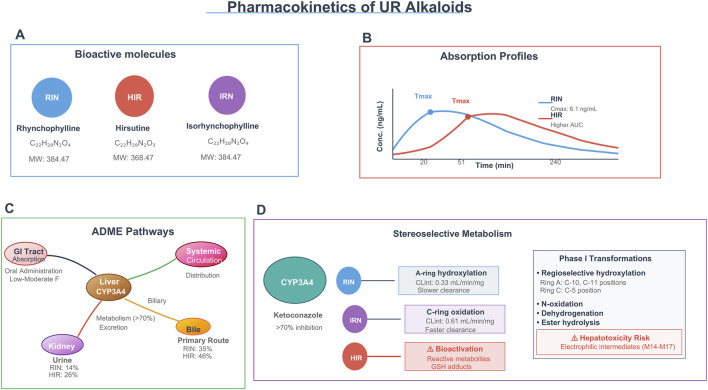
Pharmacokinetics of UR alkaloids: molecules, absorption, ADME, and stereoselective metabolism. Note: This figure overviews the pharmacokinetics of UR alkaloids: their chemical identities **(A)**, absorption profiles **(B)**, ADME pathways involving oral absorption, CYP3A4-mediated hepatic metabolism, and biliary/renal excretion **(C)**, and stereoselective metabolism (including HIR’s bioactivation to hepatotoxic intermediates) **(D)**.

Although UR is generally considered safe in traditional use, limited toxicological data exist. Preclinical studies show no acute toxicity in rats at doses up to 5 g/kg, with no significant changes in body weight, organ histology, or blood parameters (Lim and Lee, 2022). However, high doses may cause hepatotoxicity due to CYP3A4-mediated reactive metabolites from alkaloids like hirsutine (Guo et al., 2023). Adverse effects in humans are rare but include gastrointestinal complaints (nausea, diarrhea), renal effects, and neuropathy (from cat’s claw, a related species). In pregnant rats, UR alkaloid extract at 100 mg/kg showed no fetal toxicity but reduced inflammation (Wu and Xiao, 2019). Long-term use requires monitoring for herb-drug interactions and potential hormonal effects (Figure 6).

## 6 Discussion and limitations

UR is a perfect example of the combination of ancient ethnopharmacological knowledge with scientific validation, and it shows a great deal of therapeutic potential in the case of neurology and heart diseases. Through the analysis of the plant chemicals, the presence of diverse bioactive compounds has been revealed, the main group being the monoterpenoid indole alkaloids such as rhynchophylline and isorhynchophylline, but also triterpenoids, flavonoids, and phenolics. These compounds are the basis of the multitarget pharmacological activities of UR, which corresponds to the traditional Chinese medicinal (TCM) doctrine of supportive effects. A strong preclinical study, for example, corroborates UR’s neuroprotective roles in Alzheimer’s disease through the modulation of amyloid-β aggregation, tau hyperphosphorylation, neuroinflammation, and oxidative stress (Jiang et al., 2023; Shin et al., 2018). Also, in the case of Parkinson’s disease, UR acts on the TLR4/NF-κB/NLRP3 inflammasome and PI3K/Akt/mTOR pathways possibly with the intent to change the course of the disease (Zhang et al., 2024). In models of seizure, UR has shown comparable antiepileptic effects to that of valproic acid (Ho et al., 2014), while on the other hand its antihypertensive actions involve calcium channel antagonism, α1-adrenoreceptor blockade, and endothelium-dependent vasodilation (Li et al., 2018; Loh et al., 2017). The new findings also point to the immunomodulatory, antiviral, and metabolic effects of UR thereby indicating to the widening of its usage to areas outside of traditional uses.

Despite these strengths, significant limitations hinder UR’s clinical translation. Preclinical studies conducted on rats showed that even at a dose of 5 g/kg, UR does not cause any acute toxicity; the rats showed no increase or change of any kind in their body weight, the histology of their organs was normal, and there was no change in the blood parameters (Lim and Lee, 2022). On the other hand, the administration of high doses might lead to the metabolism of the alkaloids by the liver (CYP3A4) resulting in reactive metabolites carrying hirsutine toxicity (Guo et al., 2023). Adverse reactions in humans are infrequent, comprising mainly gastrointestinal disorders (nausea, diarrhea), renal effects, and neuropathy; these adverse effects are often extrapolated from related species like cat’s claw. In pregnant rats, the administration of UR alkaloids at 100 mg/kg did not result in fetal toxicity but instead the rats experienced a reduction in inflammation (Wu and Xiao, 2019); however, long-term use is still a matter of concern because of potential hormonal changes and drug-herb interactions that require constant monitoring.

Clinical evidence is particularly constrained, drawn from small-scale Asian studies with methodological flaws. Regarding the treatment of hypertension, the reports from the TCM-derived reviewed studies indicated an 83% effective rate in mild-to-moderate cases through TCM formulations, but these studies were not controlled and leaned heavily on traditional accounts. The results of a randomized trial of Tianma-Gouteng Yin gave the picture of a blood pressure decrease, with low allocation bias but high detection bias due to blinding being absent (Tai et al., 2020). Neurological indications such as those of epilepsy and Alzheimer’s hinge on anecdotal evidence with no large randomized controlled trials conducted. We are looking at a situation where the probability of bias is very high because of the small samples (n < 100), inadequate blinding, and the publication bias that favors the positive outcomes, so we need larger, standardized international trials.

In a way, the multi-target profile of UR is advantageous when compared to the traditional single-mechanism drugs, probably having fewer adverse effects. When it comes to treating high blood pressure, UR’s blocking of calcium and its vasodilatory action resemble those of amlodipine but may also lead to the lesser problem of edema; however, its potency still cannot match that of ACE inhibitors and its safety is still not established (Zhou and Zhou, 2010). UR in the case of Alzheimer’s disease is like donepezil with respect to Aβ binding and tau phosphorylation but at least provides wider neuroprotection without causing nausea related to cholinergic activity. In the case of Parkinson’s, UR’s anti-inflammatory action works in parallel with levodopa’s but has not been proven to possess the disease-modifying potential (Zeng et al., 2021b). UR could be the case for adjunct-treatment with the hope of better tolerability, yet it is absolutely necessary to conduct head-to-head trials in this regard to prove the claimed superiority.

The alkaloid variability (e.g., rhynchophylline 0.1%–1%) from various plant parts, geographical areas, and harvesting times is one of the major reasons quality control and standardization are each time hard tasks (Fu et al., 2023; Song et al., 2022). Among others, the main recommendations comprise HPLC-MS quantification, cutting at autumn for peak alkaloids, and choosing hooks for higher content, along with Good Agricultural Practices for chemical-free crop cultivation. It is metabolic differences (e.g., faster rodent CYP3A4), dose scaling (human-equivalent doses may be toxic), and model fidelity (e.g., MPTP not fully replicating human Parkinson’s) that limit the extrapolation of preclinical findings to humans. The overestimation of benefits is a consequence of these factors that emphasize the necessity of human-relevant studies.

Economically, UR is a high-value crop with annual usage over 5,000 tons in China (Wang et al., 2021), cultivated on subtropical mountain slopes in regions like Guangxi. However, sustainability is threatened by soil erosion and heavy metal accumulation, with recommendations for biomass ash as a soil conditioner to boost water retention and microbial diversity, fostering eco-friendly practices. Regulatory status varies: China’s Pharmacopoeia recognizes five *Uncaria* species but advocates standardizing to UR for consistency. In the US (FDA) and EU (EMA), related species like cat’s claw are dietary supplements without approved drug status or specific UR monographs, requiring Generally Recognized as Safe designation for food use. These disparities highlight the need for global harmonization to support broader adoption.

## 7 Conclusion and future perspectives

The role that UR assumes as the foundation for traditional medicines and today’s therapeutic agents draws attention to how the two medicine systems intersect; preclinical findings provide insights to support the therapeutic use of UR in neurology and cardiology. Although scientific findings provide insight into the therapeutic value of UR, issues related to UR’s standardization, bioavailability, and clinical validation hinder achieving its potential as a therapeutic agent. The transition of UR from an ancient, traditional medicine to an evidence-based, modern therapeutic represents a significant opportunity to create evidence-based standards for traditional medicines and support public health as neurodegenerative diseases and cardiovascular diseases increase. By integrating knowledge obtained through the disciplines of both ancient and modern sciences, we can create a guide for future medicinal plants and create modern therapeutic standards.

Future research perspectives should prioritize the development of precise, actionable frameworks to facilitate the clinical translation of UR. To this end, a stepwise translational framework is proposed, integrating preclinical optimization, pharmacokinetic enhancement, safety validation, efficacy confirmation, and sustainability-driven regulatory advancement, as detailed below:

First, preclinical optimization should focus on standardizing UR extracts through HPLC-MS-guided purification protocols. To minimize batch-to-batch variability, the framework recommends prioritizing autumn-harvested UR hooks and implementing Good Agricultural Practices (GAP)-compliant cultivation strategies, which lay a foundational basis for subsequent translational research. Second, pharmacokinetic properties of UR should be enhanced *via* nanoformulation technologies or prodrug modification, with the target of achieving a bioavailability exceeding 20%. Validation of these optimized formulations should be conducted using human hepatocyte models to evaluate and mitigate potential interactions with cytochrome P450 3A4 (CYP3A4), a key consideration for clinical applicability. Third, comprehensive safety profiling is essential for clinical translation. This includes conducting long-term toxicity studies in non-rodent models (e.g., non-human primates) to complement traditional rodent-based assessments, followed by Phase I clinical trials (sample size: 20–50 participants). These trials should focus on establishing safe dosing regimens across diverse populations, with particular attention to hepatotoxicity risks and potential herb-drug interactions—two critical safety concerns in herbal medicine translation. Fourth, efficacy validation requires large-scale, multi-center randomized controlled trials with a minimum sample size of 200 participants, focusing on UR’s therapeutic potential in hypertension and Alzheimer’s disease. Trial designs should compare UR monotherapy or classic herbal combinations (e.g., Tianma-Gouteng Yin) against placebo or standard-of-care interventions. Key endpoints should include clinically relevant outcomes such as blood pressure reduction (for hypertension) or cognitive function scores (for Alzheimer’s disease), with the integration of neuroprotective biomarkers to provide mechanistic insights into therapeutic effects. Fifth, advancing sustainability and regulatory acceptance is integral to long-term translational success. This involves advocating for the establishment of UR standards in the World Health Organization (WHO) or international pharmacopoeias, alongside conducting eco-friendly cultivation trials—for instance, utilizing soil conditioners to mitigate erosion and promote sustainable agricultural practices. Additionally, efforts should be made to pursue Generally Recognized As Safe (GRAS) certification or novel food approvals in Western markets to expand UR’s global clinical application.

## References

[B1] Bai H. Yun J. Wang Z. Ma Y. Liu W. (2024). Population pharmacokinetics study of tacrolimus in liver transplant recipients: a comparison between patients with or without liver cancer before surgery. Front. Pharmacol. 15, 1449535. 10.3389/fphar.2024.1449535 39257396 PMC11385303

[B2] Castro A. A. Nunes R. Carvalho L. R. Targueta C. P. Dos Santos Braga-Ferreira R. de Melo-Ximenes A. A. (2023). Chloroplast genome characterization of *Uncaria guianensis* and *Uncaria tomentosa* and evolutive dynamics of the Cinchonoideae subfamily. Sci. Rep. 13, 8390. 10.1038/s41598-023-34334-1 37225737 PMC10209157

[B3] Chen X. X. Leung G. P. Zhang Z. J. Xiao J. B. Lao L. X. Feng F. (2017). Proanthocyanidins from *Uncaria rhynchophylla* induced apoptosis in MDA-MB-231 breast cancer cells while enhancing cytotoxic effects of 5-fluorouracil. Food Chem. Toxicol. 107, 248–260. 10.1016/j.fct.2017.07.012 28689063

[B4] Chen L. Liu Y. Xie J. (2024). The beneficial pharmacological effects of *Uncaria rhynchophylla* in neurodegenerative diseases: focus on alkaloids. Front. Pharmacol. 15, 1436481. 10.3389/fphar.2024.1436481 39170707 PMC11336414

[B5] Choi D. Y. Huh J. E. Lee J. D. Cho E. M. Baek Y. H. Yang H. R. (2005). *Uncaria rhynchophylla* induces angiogenesis *in vitro*and *in vivo* . Biol. Pharm. Bull. 28 (12), 2248–2252. 10.1248/bpb.28.2248 16327159

[B6] Eckert G. P. (2010). Traditional used plants against cognitive decline and Alzheimer disease. Front. Pharmacol. 1, 138. 10.3389/fphar.2010.00138 21833177 PMC3153012

[B7] Fu Q. Dong W. Ge D. Ke Y. Jin Y. (2023). Supercritical fluid-based method for selective extraction and analysis of indole alkaloids from *Uncaria rhynchophylla* . J. Chromatogr. A 1710, 464410. 10.1016/j.chroma.2023.464410 37776825

[B8] Fujiwara H. Iwasaki K. Furukawa K. Seki T. He M. Maruyama M. (2006). *Uncaria rhynchophylla*, a Chinese medicinal herb, has potent antiaggregation effects on Alzheimer’s beta-amyloid proteins. J. Neurosci. Res. 84 (2), 427–433. 10.1002/jnr.20891 16676329

[B9] Gao L. Kong X. Wu W. Feng Z. Zhi H. Zhang Z. (2022). Dissecting the regulation of arachidonic acid metabolites by *Uncaria rhynchophylla* (Miq.) Miq. in spontaneously hypertensive rats and the predictive target sEH in the anti-hypertensive effect based on metabolomics and molecular docking. Front. Pharmacol. 13, 909631. 10.3389/fphar.2022.909631 35712719 PMC9196077

[B10] Geng C. A. Yang T. H. Huang X. Y. Ma Y. B. Zhang X. M. Chen J. J. (2019). Antidepressant potential of *Uncaria rhynchophylla* and its active flavanol, catechin, targeting melatonin receptors. J. Ethnopharmacol. 232, 39–46. 10.1016/j.jep.2018.12.013 30543912

[B11] Geng H. Chen X. Wang C. (2021). Systematic elucidation of the pharmacological mechanisms of rhynchophylline for treating epilepsy *via* network pharmacology. BMC Complement. Med. Ther. 21 (1), 9. 10.1186/s12906-020-03178-x 33407404 PMC7788712

[B12] Guo Q. Ma X. Wei S. Qiu D. Wilson I. W. Wu P. (2014). *De novo* transcriptome sequencing and digital gene expression analysis predict biosynthetic pathway of rhynchophylline and isorhynchophylline from *Uncaria rhynchophylla*, a non-model plant with potent anti-Alzheimer’s properties. BMC Genomics 15 (1), 676. 10.1186/1471-2164-15-676 25112168 PMC4143583

[B13] Guo Y. Lv H. Lv J. Jiang Z. (2023). Metabolite profiling and identification of enzymes responsible for the metabolism of hirsutine, a major alkaloid from *Uncaria rhynchophylla* . Xenobiotica 53, 474–483. 10.1080/00498254.2023.2269417 37819730

[B14] Ha T. Kang B. Kim M. S. Chu J. W. Kim K. Yoon W. (2025). *Uncaria rhynchophylla* and hirsuteine as TRPV1 agonists inducing channel desensitization. J. Ethnopharmacol. 337, 118869. 10.1016/j.jep.2024.118869 39353479

[B15] Hishiki T. Kato F. Tajima S. Toume K. Umezaki M. Takasaki T. (2017). Hirsutine, an indole alkaloid of *Uncaria rhynchophylla*, inhibits late step in dengue virus life cycle. Front. Microbiol. 8, 1674. 10.3389/fmicb.2017.01674 28912773 PMC5582420

[B16] Ho T. Y. Tang N. Y. Hsiang C. Y. Hsieh C. L. (2014). *Uncaria rhynchophylla* and rhynchophylline improved kainic acid-induced epileptic seizures *via* IL-1β and brain-derived neurotrophic factor. Phytomedicine 21 (6), 893–900. 10.1016/j.phymed.2014.01.011 24636743

[B17] Hou W. C. Lin R. D. Chen C. T. Lee M. H. (2005). Monoamine oxidase B (MAO-B) inhibition by active principles from *Uncaria rhynchophylla* . J. Ethnopharmacol. 100 (1–2), 216–220. 10.1016/j.jep.2005.03.017 15890481

[B18] Hou X. Liang X. Zhao X. Shi Y. Zhuo F. Tong X. (2025). *Uncaria rhynchophylla* alkaloid extract exerts neuroprotective activity against Parkinson’s disease *via* activating mitophagy with the involvement of UCHL1. J. Ethnopharmacol. 338, 119009. 10.1016/j.jep.2024.119009 39471877

[B19] Hsieh C. L. Chen M. F. Li T. C. Li S. C. Tang N. Y. Hsieh C. T. (1999). Anticonvulsant effect of *Uncaria rhynchophylla* (Miq) Jack. in rats with kainic acid-induced epileptic seizure. Am. J. Chin. Med. 27 (2), 257–264. 10.1142/S0192415X9900029X 10467459

[B20] Hsieh C. L. Ho T. Y. Su S. Y. Lo W. Y. Liu C. H. Tang N. Y. (2009). *Uncaria rhynchophylla*and Rhynchophylline inhibit c-Jun N-terminal kinase phosphorylation and nuclear factor-kappaB activity in kainic acid-treated rats. Am. J. Chin. Med. 37 (2), 351–360. 10.1142/S0192415X09006898 19507277

[B21] Hsu H. C. Tang N. Y. Liu C. H. Hsieh C. L. (2013). Antiepileptic effect of *Uncaria rhynchophylla* and Rhynchophylline involved in the initiation of c-Jun N-Terminal kinase phosphorylation of MAPK signal pathways in acute seizures of kainic acid-treated rats. Evidence-Based Complement. Altern. Med. 2013, 961289. 10.1155/2013/961289 24381640 PMC3867957

[B22] Hu S. Mak S. Zuo X. Li H. Wang Y. Han Y. (2018). Neuroprotection against MPP(+)-Induced cytotoxicity through the activation of PI3-K/Akt/GSK3β/MEF2D signaling pathway by Rhynchophylline, the major tetracyclic oxindole alkaloid isolated from *Uncaria rhynchophylla* . Front. Pharmacol. 9, 768. 10.3389/fphar.2018.00768 30072894 PMC6060423

[B23] Hu W. Li M. Sun W. Li Q. Xi H. Qiu Y. (2022). Hirsutine ameliorates hepatic and cardiac insulin resistance in high-fat diet-induced diabetic mice and *in vitro* models. Pharmacol. Res. 177, 105917. 10.1016/j.phrs.2021.105917 34597809

[B24] Huang K. Chen X. Li S. Zhang X. Zhang Y. Zhang Y. (2025). Indole alkaloids from *Uncaria rhynchophylla* and their inhibitory activities against α-glucosidase. Phytochemistry 236, 114490. 10.1016/j.phytochem.2025.114490 40147593

[B25] Jiang S. Yang X. Wang Z. Gan C. Huang J. Sun J. (2022). Biotransformation and pharmacokinetic studies of four alkaloids from *Uncaria rhynchophylla*in rat plasma by ultra-performance liquid chromatography with tandem mass spectrometry. J. Pharm. Biomed. Analysis 218, 114858. 10.1016/j.jpba.2022.114858 35691093

[B26] Jiang S. Borjigin G. Sun J. Li Q. Wang Q. Mu Y. (2023). Identification of *Uncaria rhynchophylla* in the potential treatment of Alzheimer’s disease by integrating virtual screening and *in vitro* validation. Int. J. Mol. Sci. 24 (20), 15457. 10.3390/ijms242015457 37895137 PMC10607254

[B27] Jung T. Y. Pham T. N. Umeyama A. Shoji N. Hashimoto T. Lee J. J. (2010). Ursolic acid isolated from *Uncaria rhynchophylla* activates human dendritic cells *via* TLR2 and/or TLR4 and induces the production of IFN-gamma by CD4+ naive T cells. Eur. J. Pharmacol. 643 (2–3), 297–303. 10.1016/j.ejphar.2010.06.030 20599915

[B28] Kim T. J. Lee J. H. Lee J. J. Yu J. Y. Hwang B. Y. Ye S. K. (2008). Corynoxene isolated from the hook of *Uncaria rhynchophylla* inhibits rat aortic vascular smooth muscle cell proliferation through the blocking of extracellular signal regulated kinase 1/2 phosphorylation. Biol. Pharm. Bull. 31 (11), 2073–2078. 10.1248/bpb.31.2073 18981576

[B29] Kim J. H. Bae C. H. Park S. Y. Lee S. J. Kim Y. (2010). *Uncaria rhynchophylla* inhibits the production of nitric oxide and interleukin-1β through blocking nuclear factor κB, Akt, and mitogen-activated protein kinase activation in macrophages. J. Med. Food 13 (5), 1133–1140. 10.1089/jmf.2010.1128 20828308

[B30] Kim K. S. Pham T. N. Jin C. J. Umeyama A. Shoji N. Hashimoto T. (2011). Uncarinic acid C isolated from *Uncaria rhynchophylla* induces differentiation of Th1-promoting dendritic cells through TLR4 signaling. Biomark. Insights 6, 27–38. 10.4137/BMI.S6441 21499439 PMC3076018

[B31] Kim S. Nam Y. Shin S. J. Prajapati R. Shin S. M. Kim M. J. (2022). Dual modulators of aggregation and dissociation of amyloid beta and tau: *in vitro, in vivo,*and *in silico*studies of *Uncaria rhynchophylla* and its bioactive components. Biomed. Pharmacother. 156, 113865. 10.1016/j.biopha.2022.113865 36242849

[B32] Koseki Y. Nishimura H. Asano R. Aoki K. Shiyu L. Sugiyama R. (2025). Isolation of new indole alkaloid triglucoside from the aqueous extract of *Uncaria rhynchophylla* . J. Nat. Med. 79 (1), 28–35. 10.1007/s11418-024-01836-9 39174720 PMC11735493

[B33] Kuang Y. Zhu M. Gu H. Tao Y. Huang H. Chen L. (2024). Alkaloids in *Uncaria rhynchophylla*improves AD pathology by restraining CD4(+) T cell-mediated neuroinflammation *via* inhibition of glycolysis in APP/PS1 mice. J. Ethnopharmacol. 331, 118273. 10.1016/j.jep.2024.118273 38703874

[B34] Lan Y. L. Zhou J. J. Liu J. Huo X. K. Wang Y. L. Liang J. H. (2018). *Uncaria rhynchophylla*ameliorates Parkinson’s disease by inhibiting HSP90 expression: insights from quantitative proteomics. Cell. Physiology Biochem. 47 (4), 1453–1464. 10.1159/000490837 29940559

[B35] Lee J. (2024). Triterpene esters from *Uncaria rhynchophylla* hooks as potent HIV-1 protease inhibitors and their molecular docking study. Sci. Rep. 14 (1), 31576. 10.1038/s41598-024-76551-2 39738211 PMC11686156

[B36] Lee J. S. Yang M. Y. Yeo H. Kim J. Lee H. S. Ahn J. S. (1999). Uncarinic acids: phospholipase Cγ1 inhibitors from hooks of *Uncaria rhynchophylla* . Bioorg. Med. Chem. Lett. 9 (10), 1429–1432. 10.1016/S0960-894X(99)00211-5 10360750

[B37] Lee J. S. Kim J. Kim B. Y. Lee H. S. Ahn J. S. Chang Y. S. (2000). Inhibition of phospholipase Cγ1 and cancer cell proliferation by triterpene esters from *Uncaria rhynchophylla* . J. Nat. Prod. 63 (6), 753–756. 10.1021/np990478k 10869194

[B38] Lei S. Lu J. Cheng A. Hussain Z. Tidgewell K. Zhu J. (2023). Identification of PXR activators from *Uncaria rhynchophylla* (Gou Teng) and *Uncaria tomentosa* (Cat’s Claw). Drug Metabolism Dispos. 51 (5), 629–636. 10.1124/dmd.122.001234 36797057 PMC10158501

[B39] Leung A. Y. (2006). Traditional toxicity documentation of Chinese Materia medica–an overview. Toxicol. Pathol. 34 (4), 319–326. 10.1080/01926230600773958 16787890

[B40] Li Y. Yang W. Zhu Q. Yang J. Wang Z. (2015). Protective effects on vascular endothelial cell in N’-nitro-L-arginine (L-NNA)-induced hypertensive rats from the combination of effective components of *Uncaria rhynchophylla*and Semen Raphani. Biosci. Trends 9 (4), 237–244. 10.5582/bst.2015.01087 26355225

[B41] Li R. Cheng J. Jiao M. Li L. Guo C. Chen S. (2017). New phenylpropanoid-substituted flavan-3-ols and flavonols from the leaves of *Uncaria rhynchophylla* . Fitoterapia 116, 17–23. 10.1016/j.fitote.2016.11.005 27847306

[B42] Li T. Xu K. Che D. Huang Z. Jahan N. Wang S. (2018). Endothelium-independent vasodilator effect of isocorynoxeine *in vitro* isolated from the hook of *Uncaria rhynchophylla* (Miquel). Naunyn-Schmiedeberg’s Archives Pharmacol. 391 (11), 1285–1293. 10.1007/s00210-018-1536-y 30073385

[B43] Li H. J. Wei W. L. Li Z. W. Yao C. L. Wang M. Y. Zhang J. Q. (2020). Systematic comparison of metabolic differences of *Uncaria rhynchophylla*in rat, mouse, dog, pig, monkey and human liver microsomes. Anal. Bioanal. Chem. 412 (28), 7891–7897. 10.1007/s00216-020-02922-z 32888045

[B44] Li S. Zhang Y. Guo Y. Yang L. Wang Y. (2020). Monpa, memory, and change: an ethnobotanical study of plant use in Medog County, South-East Tibet, China. J. Ethnobiol. Ethnomedicine 16 (1), 5. 10.1186/s13002-020-0355-7 32000826 PMC6993401

[B45] Li H. Wei W. Li Z. Wang M. Wei X. Cheng M. (2021a). An enhanced strategy integrating offline two-dimensional separation with data independent acquisition mode and deconvolution: characterization of metabolites of *Uncaria rhynchophylla*in rat plasma as a case. J. Chromatogr. B 1181, 122917. 10.1016/j.jchromb.2021.122917 34509821

[B46] Li R. F. Guo Q. L. Zhu C. G. Xu C. B. Wei Y. Z. Cai J. (2021b). Minor triterpenes from an aqueous extract of the hook-bearing stem of *Uncaria rhynchophylla* . J. Asian Nat. Prod. Res. 23 (4), 307–317. 10.1080/10286020.2020.1870961 33506714

[B47] Li H. Wang L. Zhang L. Liu J. Zhang H. Wang D. (2023). Study on material basis and anti-hypertensive metabolomics of different extraction methods of the *Uncaria rhynchophylla* Scrophularia formula. J. Pharm. Biomed. Analysis 233, 115464. 10.1016/j.jpba.2023.115464 37209496

[B48] Liang J. H. Luan Z. L. Tian X. G. Zhao W. Y. Wang Y. L. Sun C. P. (2019). Uncarialins A-I, monoterpenoid indole alkaloids from *Uncaria rhynchophylla*as natural agonists of the 5-HT(1A) receptor. J. Nat. Prod. 82 (12), 3302–3310. 10.1021/acs.jnatprod.9b00532 31789520

[B49] Lim H. B. Lee H. R. (2022). Safety and biological activity evaluation of *Uncaria rhynchophylla* ethanolic extract. Drug Chem. Toxicol. 45 (2), 907–918. 10.1080/01480545.2020.1786581 32693641

[B50] Lin Y. W. Hsieh C. L. (2011). Oral *Uncaria rhynchophylla* (UR) reduces kainic acid-induced epileptic seizures and neuronal death accompanied by attenuating glial cell proliferation and S100B proteins in rats. J. Ethnopharmacol. 135 (2), 313–320. 10.1016/j.jep.2011.03.018 21402140

[B51] Liu C. H. Lin Y. W. Tang N. Y. Liu H. J. Hsieh C. L. (2012). Neuroprotective effect of *Uncaria rhynchophylla* in kainic acid-induced epileptic seizures by modulating hippocampal mossy fiber sprouting, neuron survival, astrocyte proliferation, and S100B expression. Evidence-Based Complement. Altern. Med. 2012, 194790. 10.1155/2012/194790 21837247 PMC3151516

[B52] Liu L. F. Song J. X. Lu J. H. Huang Y. Y. Zeng Y. Chen L. L. (2015). Tianma Gouteng Yin, a traditional Chinese Medicine decoction, exerts neuroprotective effects in animal and cellular models of Parkinson’s disease. Sci. Rep. 5, 16862. 10.1038/srep16862 26578166 PMC4649620

[B53] Liu X. Y. Tong X. N. Liang X. M. Guo Q. Tu P. F. Zhang Q. Y. (2024). Triterpenoids from the hook-bearing stems of *Uncaria rhynchophylla* . J. Asian Nat. Prod. Res. 26 (6), 747–755. 10.1080/10286020.2024.2313542 38379373

[B54] Lo W. Y. Tsai F. J. Liu C. H. Tang N. Y. Su S. Y. Lin S. Z. (2010). *Uncaria rhynchophylla* upregulates the expression of MIF and cyclophilin A in kainic acid-induced epilepsy rats: a proteomic analysis. Am. J. Chin. Med. 38 (4), 745–759. 10.1142/S0192415X10008214 20626060

[B55] Loh Y. C. Ch’ng Y. S. Tan C. S. Ahmad M. Asmawi M. Z. Yam M. F. (2017). Mechanisms of action of *Uncaria rhynchophylla* ethanolic extract for its vasodilatory effects. J. Med. Food 20 (9), 895–911. 10.1089/jmf.2016.3804 28771084

[B56] Lu Y. Gao X. Mohammed S. A. D. Wang T. Fu J. Wang Y. (2024). Efficacy and mechanism study of Baichanting compound, a combination of *Acanthopanax senticosus* (Rupr. and Maxim.) Harms, *Paeonia lactiflora* Pall and *Uncaria rhynchophylla* (Miq.) Miq. ex Havil, on Parkinson’s disease based on metagenomics and metabolomics. J. Ethnopharmacol. 319, 117182. 10.1016/j.jep.2023.117182 37714224

[B57] Luo X. Wang S. Li D. Wen J. Sun N. Fan G. (2023). Population pharmacokinetics of tigecycline in critically ill patients. Front. Pharmacol. 14, 1083464. 10.3389/fphar.2023.1083464 36992827 PMC10040605

[B58] Mohd Sairazi N. S. Sirajudeen K. N. S. (2020). Natural products and their bioactive compounds: neuroprotective potentials against neurodegenerative diseases. Evidence-Based Complement. Altern. Med. 2020, 6565396. 10.1155/2020/6565396 32148547 PMC7042511

[B59] Nakazawa T. Banba K. Hata K. Nihei Y. Hoshikawa A. Ohsawa K. (2006). Metabolites of hirsuteine and hirsutine, the major indole alkaloids of *Uncaria rhynchophylla* . Rats. Biol. Pharm. Bull. 29 (8), 1671–1677. 10.1248/bpb.29.1671 16880624

[B60] Ndagijimana A. Wang X. Pan G. Zhang F. Feng H. Olaleye O. (2013). A review on indole alkaloids isolated from *Uncaria rhynchophylla* and their pharmacological studies. Fitoterapia 86, 35–47. 10.1016/j.fitote.2013.01.018 23376412

[B61] Ngeyvijit J. Nuansuwan S. Phoophiboon V. (2023). CYP3A4/P-glycoprotein inhibitors related colchicine toxicity mimicking septic shock. BMJ Case Reports 16 (10), e257186. 10.1136/bcr-2023-257186 37813551 PMC10565285

[B62] Qiao Y. L. Zhou J. J. Liang J. H. Deng X. P. Zhang Z. J. Huang H. L. (2021). *Uncaria rhynchophylla* ameliorates unpredictable chronic mild stress-induced depression in mice *via* activating 5-HT(1A) receptor: insights from transcriptomics. Phytomedicine 81, 153436. 10.1016/j.phymed.2020.153436 33360346

[B63] Qin J. X. Hong Y. Zhao L. Y. Wang C. Q. Fang X. Liang S. (2024). The basic chemical substances of total alkaloids of *Uncaria rhynchophylla*and their anti-neuroinflammatory activities. J. Asian Nat. Prod. Res. 26 (6), 765–771. 10.1080/10286020.2024.2315211 38373226

[B64] Qu Z. H. Liu L. Zhang X. F. Guo D. Y. Zhai B. T. Zou J. B. (2022). Exploring the scientific rationality of the phenomenon of “different dosage forms of the same prescription” of Chinese proprietary medicine based on biopharmaceutical properties of powder and pill of chuanxiong Chatiao prescription. Front. Pharmacol. 13, 893552. 10.3389/fphar.2022.893552 35754501 PMC9218571

[B65] Rahman M. H. Bajgai J. Fadriquela A. Shama S. Trinh T. T. Akter R. (2021). Therapeutic potential of natural products in treating neurodegenerative disorders and their future prospects and challenges. Molecules 26 (17), 5327. 10.3390/molecules26175327 34500759 PMC8433718

[B66] Rao L. Chen F. Gao M. H. Tan J. J. Qu S. J. Tan C. H. (2025). Monoterpene indole glycoalkaloids from the hook-bearing branches of *Uncaria rhynchophylla* . J. Asian Nat. Prod. Res. 27 (1), 31–37. 10.1080/10286020.2024.2410460 39412426

[B67] Ren J. Q. Lei X. Q. Guo Q. L. Shi J. G. (2025). Minor indoloquinolizidine monoterpene alkaloids from an aqueous extract of the hook-bearing stem of *Uncaria rhynchophylla* . J. Asian Nat. Prod. Res. 27 (7), 983–994. 10.1080/10286020.2025.2509764 40488599

[B68] Shim J. S. Kim H. G. Ju M. S. Choi J. G. Jeong S. Y. Oh M. S. (2009). Effects of the hook of *Uncaria rhynchophylla* on neurotoxicity in the 6-hydroxydopamine model of Parkinson's disease. J. Ethnopharmacol. 126 (2), 361–365. 10.1016/j.jep.2009.08.023 19703534

[B69] Shin S. J. Jeong Y. Jeon S. G. Kim S. Lee S. K. Choi H. S. (2018). *Uncaria rhynchophylla*ameliorates amyloid beta deposition and amyloid beta-mediated pathology in 5XFAD mice. Neurochem. Int. 121, 114–124. 10.1016/j.neuint.2018.10.003 30291956

[B70] Shin M. R. Kim M. J. Lee J. A. Roh S. S. (2021). Effect of *Uncaria rhynchophylla* against thioacetamide-induced acute liver injury in rat. Can. J. Gastroenterol. Hepatol. 2021, 5581816. 10.1155/2021/5581816 34557455 PMC8455208

[B71] Song L. L. Wang Y. Xu C. B. Lei X. Q. Guo Q. L. Shi J. G. (2022). Minor monoterpene derivatives from an aqueous extract of the hook-bearing stem of *Uncaria rhynchophylla* . J. Asian Nat. Prod. Res. 24 (5), 432–444. 10.1080/10286020.2022.2061961 35435775

[B72] Tai J. Zou J. Zhang X. Wang Y. Liang Y. Guo D. (2020). Randomized controlled trials of Tianma Gouteng decoction combined with nifedipine in the treatment of primary hypertension: a systematic review and meta-analysis. Evidence-based Complementary Alternative Medicine 2020, 5759083. 10.1155/2020/5759083 32089726 PMC7029275

[B73] Tang W. Eisenbrand G. (1992). Chinese drugs of plant origin, chemistry, pharmacology and use in traditional and modern medicine. Berlin: Springer Verlag, 1065.

[B74] Tang N. Y. Liu C. H. Su S. Y. Jan Y. M. Hsieh C. T. Cheng C. Y. (2010). *Uncaria rhynchophylla* (Miq) Jack plays a role in neuronal protection in kainic acid-treated rats. Am. J. Chin. Med. 38 (2), 251–263. 10.1142/S0192415X10007828 20387223

[B75] Tang N. Y. Lin Y. W. Ho T. Y. Cheng C. Y. Chen C. H. Hsieh C. L. (2017). Long-term intake of *Uncaria rhynchophylla* reduces S100B and RAGE protein levels in kainic acid-induced epileptic seizures rats. Evidence-Based Complementary Altern. Med. 2017, 9732854. 10.1155/2017/9732854 28386293 PMC5343263

[B76] Umeyama A. Yahisa Y. Okada M. Okayama E. Uda A. Shoji N. (2010). Triterpene esters from *Uncaria rhynchophylla* drive potent IL-12-dependent Th1 polarization. J. Nat. Med. 64 (4), 506–509. 10.1007/s11418-010-0438-1 20585989

[B77] Wang J. Feng B. Yang X. Liu W. Liu Y. Zhang Y. (2013). Tianma gouteng yin as adjunctive treatment for essential hypertension: a systematic review of randomized controlled trials. Evidence-Based Complementary Altern. Med. 2013, 706125. 10.1155/2013/706125 23710230 PMC3655574

[B78] Wang X. Qiao Z. Liu J. Zheng M. Liu W. Wu C. (2018). Stereoselective *in vitro*metabolism of rhynchophylline and isorhynchophylline epimers of *Uncaria rhynchophylla* in rat liver microsomes. Xenobiotica 48 (10), 990–998. 10.1080/00498254.2017.1390627 28990840

[B79] Wang Q. Zhao L. Gao C. Zhao J. Ren Z. Shen Y. (2021). Ethnobotanical study on herbal market at the Dragon Boat Festival of Chuanqing people in China. J. Ethnobiol. Ethnomedicine 17 (1), 19. 10.1186/s13002-021-00447-y 33757555 PMC7985747

[B80] Wei F. T. Song L. L. Ren J. Q. Lei X. Q. Guo Q. L. Shi J. G. (2025). Minor lactones from a water decoction of the hook-bearing stem of *Uncaria rhynchophylla* . J. Asian Nat. Prod. Res. 27 (11), 1630–1640. 10.1080/10286020.2025.2525322 40658509

[B81] Wu L. Z. Xiao X. M. (2019). Evaluation of the effects of *Uncaria rhynchophylla*alkaloid extract on LPS-induced preeclampsia symptoms and inflammation in a pregnant rat model. Braz. J. Med. Biol. Res. 52 (6), e8273. 10.1590/1414-431X20198273 31116257 PMC6526749

[B82] Wu Y. T. Lin L. C. Tsai T. H. (2014). Determination of rhynchophylline and hirsutine in rat plasma by UPLC-MS/MS after oral administration of *Uncaria rhynchophylla* extract. Biomed. Chromatogr. 28 (3), 439–445. 10.1002/bmc.3052 24122787

[B83] Wu Z. F. Wang Y. Q. Wan N. Ke G. Yue P. F. Chen H. (2015). Structural stabilities and transformation mechanism of rhynchophylline and isorhynchophylline by ultra performance liquid chromatography/time-of-flight mass spectrometry (UPLC/Q-TOF-MS). Molecules 20 (8), 14849–14859. 10.3390/molecules200814849 26287142 PMC6331816

[B84] Xian Y. F. Lin Z. X. Zhao M. Mao Q. Q. Ip S. P. Che C. T. (2011). *Uncaria rhynchophylla* ameliorates cognitive deficits induced by D-galactose in mice. Planta Medica 77 (18), 1977–1983. 10.1055/s-0031-1280125 21858756

[B85] Xian Y. F. Lin Z. X. Mao Q. Q. Hu Z. Zhao M. Che C. T. (2012). Bioassay-guided isolation of neuroprotective compounds from *Uncaria rhynchophylla* against beta-amyloid-induced neurotoxicity. Evidence-Based Complementary Altern. Med. 2012, 802625. 10.1155/2012/802625 22778778 PMC3388340

[B86] Xie L. Wang T. Lin S. Lu Z. Wang Y. Shen Z. (2022). *Uncaria rhynchophylla* attenuates angiotensin II-induced myocardial fibrosis *via* suppression of the RhoA/ROCK1 pathway. Biomed. Pharmacother. 146, 112607. 10.1016/j.biopha.2021.112607 35062072

[B87] Xiong Y. Long C. (2020). An ethnoveterinary study on medicinal plants used by the Buyi people in Southwest Guizhou, China. J. Ethnobiol. Ethnomedicine 16 (1), 46. 10.1186/s13002-020-00396-y 32807192 PMC7433110

[B88] Xu Q. Q. Shaw P. C. Hu Z. Yang W. Ip S. P. Xian Y. F. (2021). Comparison of the chemical constituents and anti-Alzheimer’s disease effects of *Uncaria rhynchophylla* and *Uncaria tomentosa* . Chin. Med. 16 (1), 110. 10.1186/s13020-021-00514-2 34706756 PMC8555092

[B89] Xu Y. Wang R. Hou T. Li H. Han Y. Li Y. (2023). Uncariphyllin A-J, indole alkaloids from *Uncaria rhynchophylla* as antagonists of dopamine D2 and Mu opioid receptors. Bioorg. Chem. 130, 106257. 10.1016/j.bioorg.2022.106257 36375349

[B90] Yang W. Ip S. P. Liu L. Xian Y. F. Lin Z. X. (2020). *Uncaria rhynchophylla* and its major constituents on central nervous system: a review on their pharmacological actions. Curr. Vasc. Pharmacol. 18 (4), 346–357. 10.2174/1570161117666190704092841 31272356

[B91] Yin T. Lu J. Liu Q. Zhu G. Zhang W. Jiang Z. (2021). Validated quantitative H^1^-NMR method for simultaneous quantification of indole alkaloids in *Uncaria rhynchophylla* . ACS Omega 6 (47), 31810–31817. 10.1021/acsomega.1c04464 34870003 PMC8638010

[B92] Yin T. Zhang H. Liu X. Wei D. Ren C. Cui L. (2024). Anti-hypertensive mechanisms of *Uncaria rhynchophylla-Alisma plantago-aquatica* L: an integrated network pharmacology, cluster analysis, and molecular docking approach. Front. Chem. 12, 1356458. 10.3389/fchem.2024.1356458 38496269 PMC10941343

[B93] Zeng P. Su H. F. Ye C. Y. Qiu S. W. Tian Q. (2021a). Therapeutic mechanism and key alkaloids of *Uncaria rhynchophylla*in Alzheimer's disease from the perspective of pathophysiological processes. Front. Pharmacol. 12, 806984. 10.3389/fphar.2021.806984 34975502 PMC8715940

[B94] Zeng P. Wang X. M. Ye C. Y. Su H. F. Tian Q. (2021b). The main alkaloids in *Uncaria rhynchophylla* and their anti-Alzheimer’s disease mechanism determined by a network pharmacology approach. Int. J. Mol. Sci. 22 (7), 3612. 10.3390/ijms22073612 33807157 PMC8036964

[B95] Zhang W. B. Chen C. X. Sim S. M. Kwan C. Y. (2004). *In vitro* vasodilator mechanisms of the indole alkaloids rhynchophylline and isorhynchophylline, isolated from the hook of *Uncaria rhynchophylla* (Miquel). Naunyn-Schmiedeberg's Archives Pharmacol. 369 (2), 232–238. 10.1007/s00210-003-0854-9 14668978

[B96] Zhang Y. B. Yang W. Z. Yao C. L. Feng R. H. Yang M. Guo D. A. (2014). New triterpenic acids from *Uncaria rhynchophylla*: chemistry, NO-inhibitory activity, and tandem mass spectrometric analysis. Fitoterapia 96, 39–47. 10.1016/j.fitote.2014.04.004 24727084

[B97] Zhang Q. Chen L. Hu L. J. Liu W. Y. Feng F. Qu W. (2016). Two new ortho benzoquinones from *Uncaria rhynchophylla* . Chin. J. Nat. Med. 14 (3), 232–235. 10.1016/S1875-5364(16)30021-8 27025371

[B98] Zhang C. Zhou J. Zhuo L. Zhang W. Lv L. Zhu L. (2024). The TLR4/NF-κB/NLRP3 and Nrf2/HO-1 pathways mediate the neuroprotective effects of alkaloids extracted from *Uncaria rhynchophylla*in Parkinson’s disease. J. Ethnopharmacol. 333, 118391. 10.1016/j.jep.2024.118391 38797377

[B99] Zheng M. Chen M. Liu C. Fan Y. Shi D. (2021). Alkaloids extracted from *Uncaria rhynchophylla*demonstrate neuroprotective effects in MPTP-induced experimental Parkinsonism by regulating the PI3K/Akt/mTOR signaling pathway. J. Ethnopharmacol. 266, 113451. 10.1016/j.jep.2020.113451 33049346

[B100] Zhong Y. Wang H. Wei Q. Cao R. Zhang H. He Y. (2019). Combining DNA barcoding and HPLC fingerprints to trace species of an important Traditional Chinese Medicine Fritillariae Bulbus. Molecules 24 (18), 3269. 10.3390/molecules24183269 31500338 PMC6766824

[B101] Zhou J. Zhou S. (2010). Antihypertensive and neuroprotective activities of rhynchophylline: the role of rhynchophylline in neurotransmission and ion channel activity. J. Ethnopharmacol. 132 (1), 15–27. 10.1016/j.jep.2010.08.041 20736055

[B102] Zhou H. F. Li W. Y. Peng L. Y. Li X. N. Zuo Z. L. Zhao Q. S. (2021). Rhynchines A-E: Ca_v_3.1 calcium channel blockers from *Uncaria rhynchophylla* . Org. Lett. 23 (324), 9463–9467. 10.1021/acs.orglett.1c03641 34818888

[B103] Zhou H. F. Li W. Y. Wu Q. Ren J. Peng L. Y. Li X. N. (2023). Discovery and biomimetic semisynthesis of spirophyllines A-D from *Uncaria rhynchophylla* . Org. Lett. 25 (24), 4434–4438. 10.1021/acs.orglett.3c01342 37288843

